# Heat stress-induced remodelling of lipid and microRNA networks in *Brassica napus* germinated pollen and pollen-derived small extracellular vesicles

**DOI:** 10.1093/jxb/erag303

**Published:** 2026-06-23

**Authors:** Alessia D’Agostino, Gabriele Di Marco, Chiara Pontecorvi, Chiara Suanno, Flavia Ruggirello, Giampaolo Zuccheri, Gianfranco Ulian, Giovanni Valdrè, Antonella Canini, Stefano Del Duca, Angelo Gismondi

**Affiliations:** Department of Biology, University of Rome Tor Vergata, Rome 00133, Italy; Department of Biology, University of Rome Tor Vergata, Rome 00133, Italy; Department of Biology, University of Rome Tor Vergata, Rome 00133, Italy; Department of Biological, Geological, and Environmental Sciences, University of Bologna, Bologna 40126, Italy; Department of Biological, Geological, and Environmental Sciences, University of Bologna, Bologna 40126, Italy; Department of Pharmacy and Biotechnology, University of Bologna, Bologna 40126, Italy; Department of Biological, Geological, and Environmental Sciences, University of Bologna, Bologna 40126, Italy; Department of Biological, Geological, and Environmental Sciences, University of Bologna, Bologna 40126, Italy; Department of Biology, University of Rome Tor Vergata, Rome 00133, Italy; Department of Biological, Geological, and Environmental Sciences, University of Bologna, Bologna 40126, Italy; Department of Biology, University of Rome Tor Vergata, Rome 00133, Italy; University of Nottingham, UK

**Keywords:** expressed miRNome, fatty acids, fertilization, lipid network, miRNA, oilseed rape, pollensome, pollen–stigma interaction, rapeseed, signalling molecular mediators

## Abstract

Heat stress (HS) is a primary factor limiting plant reproductive success, severely affecting pollen production and performance. In order to clarify the molecular alterations underlying HS-induced male sterility, lipidomic and small-RNA sequencing analyses were conducted on hydrated pollen, germinated pollen, pollensomes (PSs), and the vesicle-free medium of *Brassica napus* cv ‘Phoenix CL’ grown under both control and HS conditions. HS significantly reduced pollen germinability and increased PS externalization. Although particle size remained unchanged, a significant increase in PS abundance was observed in HS-derived samples. Lipid profiling revealed significant HS-induced remodelling across all samples, including an increase of saturated fatty acids in hydrated pollen and germinated pollen. Notably, triacontanoic acid, the dominant lipid in control PS, was lost under HS conditions and replaced by oleic acid. In parallel, small-RNA sequencing identified 70 miRNAs, 61 of which were differentially expressed. Hydrated pollen showed the strongest response to HS, while PS showed opposite trends, suggesting the selective retention or export of miRNAs. Among all, HS increased the levels of miR160, miR6030a, and miR319a in PS, while the release of miR399 shifted from being vesicular to non-vesicular. Target prediction revealed that these miRNAs regulate pathways associated with development, hormones, vesicles, and lipids. Overall, this study reveals that HS remodels lipid metabolism and miRNA-mediated regulation in pollen and PS, providing molecular signatures for improving crop heat tolerance.

## Teaser

Heat stress radically alters the pollen lipid profile and microRNA network in rapeseed and the identification of crucial molecular signatures provides promising markers for improving crop heat tolerance.

## Introduction

Global warming is expected to significantly affect biodiversity, species distribution, and agricultural productivity over the coming decades. The Mediterranean region is particularly vulnerable to rising temperatures (Begcy and Dresselhaus, 2018; De Ollas *et al*., 2019; IPCC, 2021).

Environmental shifts, mainly heat stress (HS), impose severe challenges to plant reproduction (Begcy and Dresselhaus, 2018; Jagadish *et al*., 2021; Zhang *et al*., 2021). In this context, microgametogenesis, the formation of mature pollen grains within the anthers, has been shown to be sensitive to HS in Spermatophytes (Sze *et al*., 2021; Zhang *et al*., 2021). High temperatures during microgametogenesis, anther dehiscence, and flower anthesis, can compromise pollen development, leading to reduced viability, germination capacity, and fertilization efficiency (Prasad *et al*., 2017; Pacini and Dolferus, 2019; Sze *et al*., 2021). Thus, crop species and varieties, which are often bred for yield rather than stress tolerance, are very susceptible to HS-induced male sterility (Prasad *et al*., 2017; Ma *et al*., 2018). In cereals, for instance, an increase in temperature of just 1 °C has been associated with a yield loss of up to a 10% (Zhang *et al*., 2021). Similar concerns are growing around legumes and oilseeds, especially rapeseed (*Brassica napus*), due to their global economic relevance and the urgent need for climate-resilient agriculture (Prasad *et al*., 2017; Lohani *et al*., 2021).

Advances in omics technologies, such as proteomics, transcriptomics, and genomics, are shedding light on the molecular pathways underlying pollen responses to HS, including changes in gene expression and protein synthesis (Sze *et al*., 2021). HS can also induce oxidative damage, which can potentially impair protein stability and enzymatic activity, critical factors in triggering stress responses. While many of the proteins involved in pollen development and pollen–pistil signalling under optimal conditions are well characterized (Aloisi *et al*., 2016; Mandrone *et al*., 2019; Yang *et al*., 2021), the regulatory role of miRNAs in these processes is largely unexplored. Despite extensive studies on pollen development under HS, the role of small RNAs in the selective loading of extracellular vesicles (pollensomes) in *Brassica* species remains largely unknown (Grant-Downton *et al*., 2009; Wei *et al*., 2011; Zhang *et al*., 2018; Sajad *et al*., 2023). Furthermore, analysis of their cytoplasmic distribution and subcellular compartmentalization is still scarce (Oliver *et al*., 2022), despite their broad molecular functions. This knowledge gap is particularly relevant because these small non-coding RNAs influence DNA expression (Zhao *et al*., 2025), modulating key developmental and physiological processes, such as morphogenesis, hormone signalling, flowering, meristem formation, and acclimation. In this context, precise estimates of the proportion of genes under the control of miRNAs remain undetermined to date. MiRNAs are transcribed by RNA polymerase II from MIR genes and incorporated into the RNA-induced silencing complex (RISC), where they guide the degradation or translational repression of the mRNA target (Dong *et al.*, 2022; Zhan and Meyers, 2023). It has also been demonstrated that miRNAs can modulate gene silencing via DNA methylation (Place *et al*., 2008; Orang *et al*., 2014; Xie *et al*., 2015). This is a particularly relevant mechanism, given that HS seems to disrupt plant methylation patterns, thereby contributing to male sterility (Ma *et al*., 2018).

Beyond their intracellular bioactivity, miRNAs can also function as intercellular signals. They can be secreted directly into the extracellular space in the form of duplexes or single-stranded mature nucleic acids, or within vesicles that act as carriers. This allows miRNAs to be transferred between cells, tissues, and even organisms (e.g. plant–plant and plant–parasite; Samad *et al*., 2021; Loreti and Perata, 2022). They have therefore been suggested to be involved in pollen–pistil communication during fertilization, a delicate process whose underlying mechanisms are not yet fully understood. Over the last decade, it has been suggested that extracellular vesicles, which are secreted by either the pistil (Safavian and Goring, 2013) or the pollen (Tian *et al*., 2021), might play a role in such crosstalk. Indeed, it has been shown that germinating pollen release small extracellular vesicles, known as pollensomes (PS), the protein composition of which has only been characterized to date in two species (Prado *et al*., 2014; Suanno *et al*., 2023), while their miRNA cargo also remains unexplored. Disruption of this communication pathway by HS could result in decreased pollen fertilization efficiency, thus contributing to HS-induced male sterility. HS could interfere with PS-mediated communication by modulating their miRNA cargo and/or modifying their lipid profile. HS has already been linked to the alteration of lipid composition in pollen membranes, which could affect male fertility (Prasad *et al*., 2017).

This study employed lipidomic and smallRNA-sequencing approaches to investigate how HS reshapes the molecular landscape of hydrated pollen, germinated pollen, and PS in *B. napus*. Our aim was to collect data on the potential roles of miRNAs and lipids in pollen–pistil interactions under normal and HS conditions, to provide new insights into the mechanisms of HS-induced sterility, and to support the development of heat-resilient crop varieties.

## Materials and methods

### Plant growth conditions, treatments, and sampling

The study was conducted on *Brassica napus* ‘Phoenix CL’, a hybrid winter variety belonging to the Clearfield weed-management system, known for its high agronomic adaptability and tolerance to various environmental stresses. Seed germination was carried out in the dark in Petri dishes with 100% relative humidity at 22±1 °C for 10 d. Once the seedlings reached the phenological stage GS05 in the BBCH scale (Lancashire *et al*., 1991), corresponding to radicle emergence, they were transferred to sterilized soil. Plants were then grown under controlled conditions in a growth chamber, with a constant temperature of 22±1 °C under a 16/8 h light/dark photoperiod (200 µmol m^−2^ s^−1^ provided by LED lamps) and a relative humidity of 60–65%. Plants were irrigated regularly with distilled water and fertilized with a granular N-P-K fertilizer (12-12-17 + 2% MgO and 20% SO_3_) for their whole life cycle (Liu *et al*., 2014; Cibils-Stewart *et al*., 2015). In this first stage, plants were supplemented with 12% N and 6% K_2_O. After reaching GS13 (∼5–6 unfolded leaves), all plants underwent a 45 d vernalization period at 4±1 °C under the same 16/8 h photoperiod. After vernalization, plants were brought back to the standard conditions described above. Upon reaching GS61 (10% of open flowers on the main inflorescence), which is the most sensitive stage to heat stress (Chen *et al*., 2021), 60 plants were randomly assigned to the heat-stress (HS) group, while 60 other plants were assigned to the control (Ctrl) group. The growing conditions remained standard for the Ctrl group, while the HS group was transferred to another climate-controlled chamber with the same light intensity and relative humidity as the Ctrl group but with different temperature conditions, as follows. A diurnal HS regime was imposed for 10 consecutive days by gradually increasing the temperature to 35±1 °C during the first 6 h of light, then maintaining this temperature for 4 h, and finally decreasing it back to 22±1 °C during the last 6 h of light (Chen *et al*., 2021). Based on preliminary tests, this 10 d stress duration was applied to induce a sustained but non-lethal reproductive stress that was capable of inducing a clear fertility-related phenotype, characterized by a reduction in pollen productivity and germinability, while ensuring the recovery of metabolically active pollen required for high-throughput analyses. After the 10 d treatment period, the HS group was brought back to the standard growth conditions. Pollen sampling started at the end of the HS treatment (10 d after reaching GS61), and was performed every other day for 4 weeks. Freshly opened flowers were sampled with tweezers, and pollen grains were immediately collected by brushing the dehisced anthers on waxed paper. Pollen was then weighed, transferred to a test tube, frozen in liquid nitrogen, and stored at −80 °C until subsequent analysis (within 1 month).

### Pollen hydration and germination treatments

Freshly collected pollen grains were micrographed under light microscopy and measured with ImageJ (Schneider *et al*., 2012). For both hydration and germination treatments, 20 mg of frozen pollen grains per sample were rehydrated in a humid chamber at 21 °C with 100% relative humidity for 30 min. The pollen viability was then assessed by MTT assays as previously described (Aloisi *et al*., 2015). Half of the samples (hydrated pollen samples, HP) were then stored at −80 °C for subsequent analyses, while the other half (germinated pollen samples, GP) were resuspended in 20 ml of germination medium (16% sucrose, 0.8 mM H_3_BO_3_, 1.6 mM CaCl_2_, 1 mM KNO_3_, and 1.65 mM Tris, dissolved in ultrapure water), and incubated at 21 °C for 2 h under gentle shaking. The germination rates and pollen-tube length of the GP samples were then assessed by light microscopy on 100 grains per sample, with three replicates. Pollen was considered germinated when the tube length was equal or greater than the grain diameter. Data were analysed in RStudio (https://posit.co/products/open-source/rstudio), testing their normal distribution using the Shapiro–Wilk test, and then comparing the HS and Ctrl treatments with Student’s *t*-test for parametric data, and Wilcoxon’s test for non-parametric data.

### Isolation of pollensomes

PS isolation from the GP samples was performed as previously described (Suanno *et al*., 2023), with slight adjustments. In brief, pollen grains were pelleted at 4500 *g* for 20 min at 4 °C and then either stored at −80 °C for further analyses or kept on ice for subsequent protein extraction. The supernatant was filtered twice through 0.22 μm syringe filters, and centrifuged at 100000 *g* for 70 min at 4 °C. The PS pellets were resuspended in 50 μl of Dulbecco’s PBS for nanoparticle tracking analysis (NTA), lipidomic, and miRNA sequencing, whilst for immunoblotting they were resuspended in 50 μl of protein extraction buffer [20 mM Tris-HCl pH 8.5, 2 mM DTT, 0.005% (v/v) Tween80, 3 mM EDTA, protease inhibitor cocktail—Merck, Italy—1:100]. The extracellular vesicle-free (EVF) supernatant was stored at −80 °C for lipidomic and miRNA sequencing or kept on ice for subsequent protein precipitation. As a negative control for the NTA, PS isolation was performed on samples of pure germination medium, while for the lipidomic and miRNomic analyses a sample of the germination medium was collected immediately after its preparation and examined for the presence of potential miRNAs and lipid molecules.

### Visualization of pollensomes

NTA was performed immediately after PS isolation, using a ZetaView MONO-NTA analyser (Particle Metrix GmbH, Germany). PS isolated from the HS and Ctrl samples were diluted in Dulbecco’s PBS to 1:1000 in order to reach a concentration suitable for particle analysis. The PS fraction isolated from negative controls was diluted to 1:200 with Dulbecco’s PBS to reach the minimum volume necessary for the NTA. Samples were analysed in ‘Size Distribution’ mode, ‘2 Cycles’, ‘11 Positions’. Finally, the average particle concentration of the negative controls was subtracted from the particle concentration of the HS and Ctrl samples. The particle sizes and concentrations of the PS fraction were tested for normality using the Shapiro-Wilk test and compared with Student’s *t*-test. For visualization by atomic force microscopy (AFM) and environmental scanning electron microscope (ESEM), samples were prepared as described by Suanno *et al*. (2023). Briefly, a freshly cleaved MICA sheet was incubated for 30 min with 0.1% (w/v) poly-L-lysine to positively charge the surface. The modified MICA was then washed three times with ultrapure water and incubated with the PS fraction of the sample or the negative control for 1 h, washed three times with ultrapure water, and then left to dry overnight under a laminar flow hood. AFM imaging was performed on the dried samples in air at ambient humidity. A Bruker Multimode 8 AFM (Nanoscope V) was used with an E-type scanner operating in ScanAsyst™ PeakForce Tapping-mode™ (at 2 kHz or 4 kHz) with ScanAsyst air probes (Bruker). Tapping amplitude, force set-point, and noise level were minimized while ensuring stable imaging. The only processing of the AFM images was for background flattening with a third-order polynomial by using the manufacturer’s software (NanoScope Analysis v.1.8). Where present, very high surface features were excluded from the flattening calculations. ESEM analysis was conducted using a ThermoScientific Quattro S instrument equipped with a field-emission gun, backscattered electron and cathodoluminescence detectors, and a silicon-drift detector for energy dispersive X-ray spectroscopy. This was performed to investigate the morphology and local chemistry of the PS fraction in the Ctrl sample, confirming that the particles visualized by AFM were consistent with extracellular vesicle (EVs). The ESEM analysis was carried out in low-vacuum conditions, with water vapour pressure inside the chamber between 100–150 Pa, an acceleration voltage of 18 kV, and a beam current of 0.15 pA. These settings were selected after preliminary calibration to ensure high-resolution imaging while preserving the form factor of the PS.

### Molecular characterization of pollensomes

To fully characterize the PS according to MISEV guidelines (Welsh *et al*., 2024), the presence of PS molecular markers (positive markers) and the absence of those from other cell compartments (negative markers) was assessed by immunoblotting. Briefly, total proteins were extracted from the pellets obtained by centrifugation at 4500 *g* by adding 300 μl of pollen extraction buffer and 20 mg of quartz powder to each sample and rupturing the cell wall using a TissueLyser III (Quiagen) for four cycles of 1 min at 30 Hz. The samples were then centrifuged at 1000 *g* for 10 min at 4 °C to discard the quartz and cell debris, and the supernatant containing the total protein lysate was collected. The EVF supernatants obtained previously were mixed with 10% TCA and incubated for 1 h at 4 °C to precipitate the proteins, followed by centrifugation at 15 000 *g* for 5 min. The pelleted proteins were washed twice with cold (−20 °C) acetone and resuspended in 50 μl protein extraction buffer. The protein contents of the total lysate, the EVF fraction, and the PS were assessed by Bradford assays. Samples of 25 μg of proteins from the total lysate, and the whole protein contents of the PS and EVF fraction were diluted in Laemmli buffer and resolved by SDS-PAGE using a 12% polyacrylamide mini-gel (Bio-Rad). Proteins were then blotted onto a nitrocellulose membrane, subjected to Ponceau staining, blocked in 5% Blotting Grade Blocker (BioRad) in TBS, and then immunoprobed for the presence of positive and negative markers. Clathrin and BRO1-domain proteins were considered as the positive markers and were detected using Anti-Clathrin heavy-chain 1,2 polyclonal antibodies (AS10 690, Agrisera) and Anti-ALIX polyclonal antibodies (ab76608, Abcam), respectively. H^+^-ATPase (marker of the plasma membrane), plant Cytochrome oxidase subunit II (marker of the mitochondria), UDP-glucose pyrophorsphorylase (cytoplasm maker), and ADP-ribolysation factor 1 (Golgi marker) were considered as the negative markers, indicating PS and EVF-fraction contamination from the cellular debris. The negative markers were detected using the following polyclonal antibodies (all Agrisera): anti-H^+^-ATPase, AS08 325; anti-COXII, AS04 053A;, anti-UGPase, AS14 2813; and anti-ARF1, AS08 325. Membranes were developed with Amersham™ ECL Prime Western Blotting Reagents (GE Healthcare) and analysed using an Azure 280 chemiluminescent imaging system (Azure Biosystems, California). Experiments were repeated three times for each target protein (Suanno *et al*., 2023).

### GC-MS analysis

Samples (20 mg) of PS and the EVF fraction obtained from HS and Ctrl plants in the HP and GP treatments, were dried in a speed-vac system (Eppendorf Concentration Plus) and then dissolved in 1 ml of chloroform/methanol extraction solvent (2:1, v/v) for 20 min under agitation in the dark at 4 °C. After centrifugation at 2000 *g* for 10 min, the supernatant was recovered, dried, and then resuspended in pure chloroform and subjected to GC-MS analysis (D’Agostino *et al*., 2024). Aliquots of 2 µl of extract were injected into a Shimadzu QP2010 system equipped with an SH-Rtx5MS column (length 30 m, diameter 0.25 mm, thickness 0.25 µm) in splitless modality. The temperature gradient was set as follows: an initial step at 50 °C for 2 min, an increase of 3 °C min^–1^ up to 200 °C, constant at 200 °C for 2 min, an increase of 4 °C min^–1^ up to 300 °C, and then constant at 300 °C for 5 min. Helium was used as the carrier gas at a constant flow of 1 ml min^–1^. The electron impact was set at 70 eV (scanning from 100–700 *m*/*z*), the ion source temperature at 250 °C, the interface temperature at 280 °C, and the solvent cut time at 6 min. All peaks from the chromatograms were analysed and each analyte was identified by comparing its MS-based molecular profile with those of pure standards registered in the National Institute of Standard and Technology Library 14 loaded in the detection software of the instrument; only identity values higher than 85% were considered acceptable. The abundance of the metabolites is reported as relative percentage values with respect to the total mixture (100%).

### MiRNA isolation and quality assessment

PS, the EVF fraction, and whole pollen grains of HP and GP samples from Ctrl and HS plants were flash-frozen in liquid nitrogen and immediately ground in 1.5 ml Eppendorf tubes to ensure rapid tissue disruption, except for the EVF samples that were already liquid. MiRNAs were then purified using MirPremier microRNA Isolation Kits (Sigma-Aldrich) following the manufacturer’s instructions. The quality and concentration of the isolated miRNAs were assessed using a NanoDrop 2000 spectrophotometer (ThermoFisher Scientific). To verify the reliability of the extraction procedure, qPCR assays were performed using miRNA-derived cDNAs as a template, according to the procedure described by Gismondi *et al*. (2017), using a StepOnePlus Real-Time PCR System (Perkin-Elmer, Applied Biosystems). In brief, 50 ng of miRNAs were reverse-transcribed into cDNA using a miRCURY LNA RT Kit (Qiagen). A synthetic spike-in control miRNA (UniSp6, Exiqon) was added to monitor potential nuclease contamination, as well as the efficiency of cDNA synthesis and subsequent PCR amplifications. In detail, the presence of UniSp6, a conserved plant miRNA (miR159a, miRBase accession: MIMAT0000177) and the plant 5S rRNA (used as positive control; *Arabidopsis thaliana*, GenBank: AB073495.1) was assessed. Negative controls, that is reactions lacking either the template (Neg1) or primers (Neg2), were also included in this phase to confirm the specificity of the amplification. PCR products were separated by electrophoresis on a 1% (w/v) agarose gel containing 10 mg ml^–1^ ethidium bromide in TAE buffer (40 mM Tris, 20 mM acetic acid, 1 mM EDTA; pH 8.5) and visualized under UV light using a ChemiDoc Imaging System (Bio-Rad). After this check, the miRNA pools extracted from the samples were subjected to high-throughput screening analysis as described below.

### Sequencing and miRNome analysis

Indexed libraries were generated using a NEXTflex Small RNA-Seq Kit v3 (Perkin Elmer), following the manufacturer’s protocol, starting from the previously isolated miRNA pools. Library quantitation was performed with both the TapeStation 4200 system (Agilent Technologies) and a Qubit Fluorometer (Invitrogen). Libraries were then pooled to ensure equimolar representation of all index-tagged samples. Sequencing was conducted on an Illumina NovaSeq 6000 System using a 75 bp single-end configuration. Raw data quality from small-RNA sequencing was assessed using FastQC (v0.12.1; https://www.bioinformatics.babraham.ac.uk/projects/fastqc/), while 3′ adapter trimming was performed using sRNAbench (v2.0) (Aparicio-Puerta *et al*., 2019). Reads between 17–30 nt were aligned to mature miRNAs in PmiREN2.0 (Guo *et al*., 2020). To reduce alignment noise due to identical miRNA sequences across species, reads were aligned selectively to Brassicaceae miRNAs, including *B. juncea* (Bju), *B. napus* (Bna), *B. nigra* (Bni), *B. oleracea* (Bol), and *B. rapa* (Bra) accessions. Residual redundancy was addressed by clustering sequences at 90% similarity using CD-HIT (v4.8.1) (Li and Godzik, 2006) and selecting a representative miRNA per cluster. In this study, miRNA names followed two conventions, as suggested by Guo *et al*. (2020): first, known and previously annotated miRNAs (as found in public databases such as miRbase) were labelled with the prefix ‘miR’ (e.g. Bju-miR158a); and second, novel or putatively identified miRNAs (predicted through computational analysis and not yet deposited in databases such as miRbase), were labelled with the prefix ‘miRN’ (e.g. Bra-miRN357a). This distinction allows for clear differentiation between conserved and newly identified miRNAs within the dataset. The expression of mature miRNAs in each library was normalized to counts per million reads (CPM) as follows: CPM = (miRNA read counts/total mapped read counts per library) × 1×10^6^. Differential expression analysis of miRNAs was performed across all samples. Differentially expressed miRNAs were defined as miRNAs showing a *z*-score ≥|1| in each respective comparison. Log_2_(fold-change) was calculated as log_2_(read count+1)/log_2_(read count+1). Heatmap visualization of miRNA expression profiles was generated using SRplot (Tang *et al*., 2023). An UpSet plot, implemented via the *ComplexHeatmap* package (Gu *et al*., 2016), was used to efficiently display the intersections of miRNAs across different sample groups. Differentially expressed miRNAs were analysed using psRNATarget (Dai *et al*., 2018) to predict their putative mRNA targets in *B. oleracea*. To infer gene function, predicted targets were mapped to one-to-one orthologues in Arabidopsis using Phytozome BioMart (Goodstein *et al*., 2012). Gene Ontology (GO) enrichment analysis was then performed using Metascape (Zhou *et al*., 2019) and clusterProfiler v4.10.1 (Wu *et al*., 2021), which identifies statistically enriched terms and clusters them based on the Kappa similarity index among gene memberships.

### Validation of miRNome data by real-time qPCR

To validate the high-throughput sequencing results, six miRNAs (miR158a, miR159a, miR161, miR172, miR289, and miR399) were quantified using real-time qPCR using the StepOnePlus Real-Time PCR System, as described above. Briefly, 50 ng of miRNA-derived cDNA were amplified in a 10 µl reaction volume containing 50% Kapa SYBR Fast qPCR Master Mix (Kapa Biosystems, Woburn, MA, USA) and 1 µl of specific LNA miRNA PCR primer sets (Qiagen). The thermal cycling conditions were: 95 °C for 10 min, followed by 45 cycles of 95 °C for 15 s, and 60 °C for 1 min. A final melting-curve analysis was performed to ensure amplicon specificity. The relative expression levels were calculated using the 2^−ΔΔ*C*T^ method, with *5S rRNA* as the internal reference gene. Experiments were repeated three times. Statistical analysis was performed using two-way ANOVA followed by Tukey’s LSD test using Microsoft Excel. Results are expressed as means (±SD) and normalized to the HP-Ctrl sample, which was set as the reference unit (100 arbitrary units, A.U.), and visualized on a log_10_ scale.

## Results

### Heat stress influences pollen production, germinability, and pollensome externalisation

**Fig. 1.**
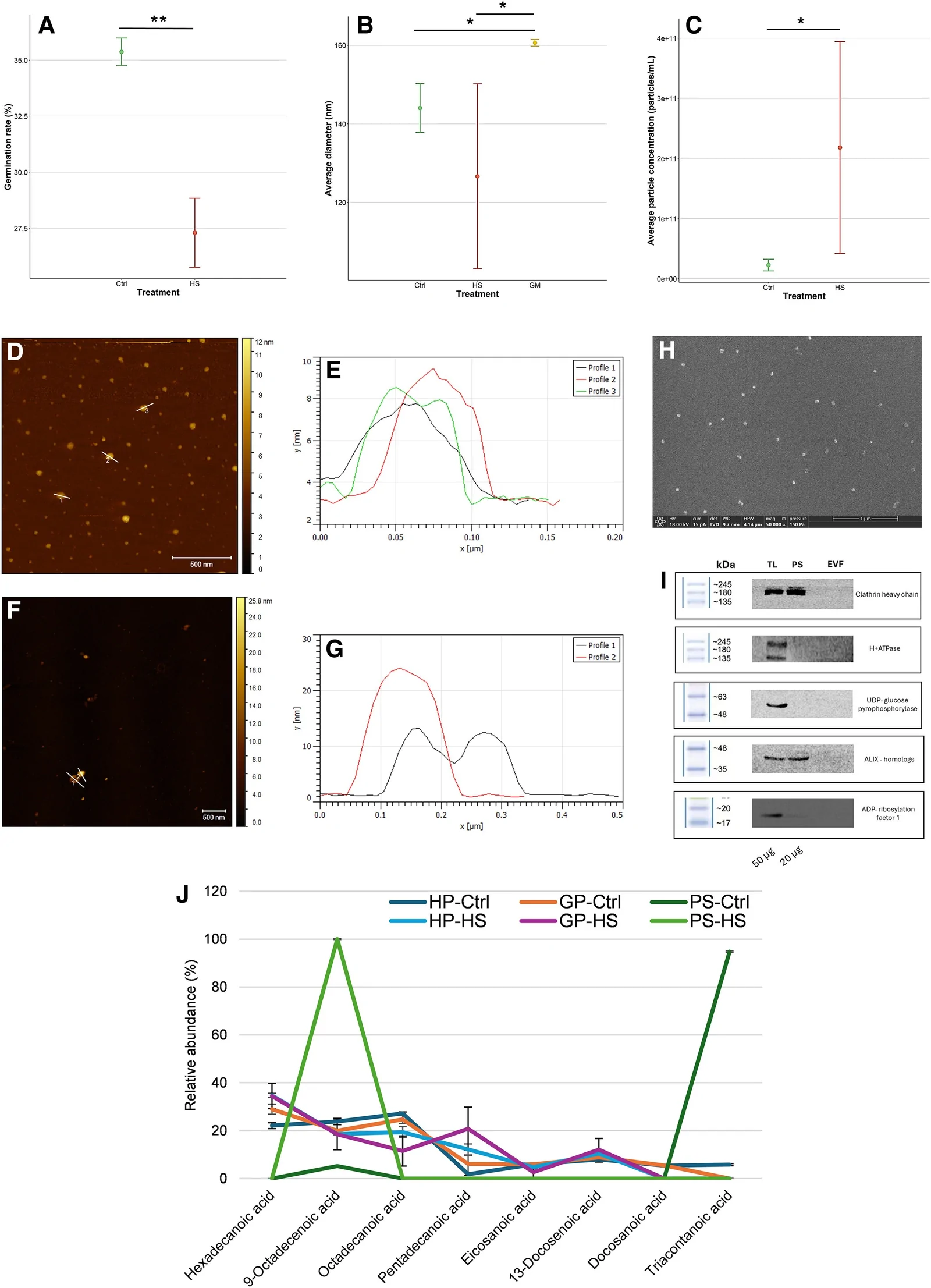
Effects of heat stress on pollen and pollensome production in *Brassica napus*. (A) *In vitro* germination rates of pollen grains from plants in the control (Ctrl) and heat-stress (HS) treatments. (B) Mean particle diameters in the pollensome (PS) fraction of the Ctrl and HS samples, and in the germination medium (GM). (C) Mean particle concentration in the PS fraction of Ctrl and HS pollen. Data are means (±SD), based on *n*=3 batches of 100 pollen grains. Significant differences between treatments were determined using either Student’s *t*-test or Wilcoxon’s test for non-parametric data: *P<0.05; **P<0.01. (D) Representative atomic force microscopy (AFM) image of the PS fraction from Ctrl plants, and (E) the topographic profiles of the three particles indicated in the image. (F) Representative AFM image of the PS fraction from HS plants, and (G) the topographic profiles of the two indicated particles, one of which displays the typical cup-shaped profile of larger extracellular vesicles. (H) A representative environmental scanning electron microscope image of the PS fraction from Ctrl plants. (I) Representative western blot analysis of marker proteins isolated from the total lysate (TL) of germinated pollen, the PS fraction, and the extracellular vesicle-free (EVF) supernatant of the extraction solution. (J) Relative abundance of fatty acids detected by GC-MS analysis in the Ctrl and HS treatments for hydrated pollen (HP), germinated pollen (GP), and the PS fraction. Data are means (±SD), *n*=3 replicates.

Heat stress dramatically affected pollen productivity, with plants in the HS treatment producing on average 1.250 mg of fresh pollen per plant compared with 3.125 mg in the Ctrl group. This reduction in pollen production probably reflects an early-stage developmental failure, as heat stress is known to trigger flower and anther abortion, thereby limiting the total number of grains produced. Microscopic analysis of freshly collected grains did not reveal any visible morphological differences between the two groups, and they showed comparable dimensions of both the polar axis (Ctrl, 34.06±2.51 µm; HS, 35.36±1.86 µm) and the equatorial axis (Ctrl, 26.01±2.19 µm; HS, 27.06±1.69 µm). The viability of freshly collected pollen was very high in both groups (98%), and also showed no significant difference. This is explained by the fact that the analysis was conducted only on the fraction of pollen that successfully reached anthesis and completed maturation. Hence, while HS reduced the overall investment in pollen production, the grains that were finally produced retained their basal metabolic activity. However, the *in vitro* germinability was affected by heat stress, with HS pollen having a mean germination rate of 27.30%, which was significantly lower than that of Ctrl pollen (35.37%; Fig. 1A). This indicated that the metabolic viability did not necessarily equate to physiological functionality, and demonstrated that the HS treatment significantly impaired the germination capacity of the surviving grains.

Nanoparticle tracking analysis revealed the presence of nanoparticles in the pollensome (PS) fraction of both HS and Ctrl pollen. The diameter of these nanoparticles did not differ significantly and ranged from 27–209 nm, with a mean value of 138 nm, and a greater variability was observed for HS samples compared with Ctrl (Fig. 1B). Nanoparticles isolated from the germination medium were larger and less variable compared to those derived from PS. According to the tracking analysis, despite the lower germination rate, the mean nanoparticle concentration in the PS fraction of the HS samples was 2.18×10^11^ particles ml^–1^, almost 10-fold higher than that of Ctrl samples (2.63×10^10^ particles ml^–1^; Fig. 1C). The nanoparticles micrographed by AFM in this study were compatible in size and shape with EVs identified and characterized previously (Bagrov *et al*., 2021; Suanno *et al*., 2023). Ctrl samples presented a higher percentage of smaller particles (Fig. 1D, E) while HS samples displayed larger particles (Fig. 1F, G), consistent with the tracking analysis. ESEM visualization performed on Ctrl samples corroborated AFM results, showing particles comparable in size and shape with EVs micrographed by electron microscopy (Fig. 1H), and the elemental analysis confirmed they were carbon-based, excluding possible contamination from PBS salts (Barranco *et al*., 2019).

Western blotting revealed the absence of molecular markers from other cell compartments in the PS fraction, thus excluding contamination from cell debris, and demonstrated the presence of both the clathrin heavy-chain and of a plant homolog of ALIX with a molecular weight of ∼40 KDa. A BLAST search confirmed the presence of a Bro-1 domain-containing protein (encoded by the gene A0A078G7V0_BRANA) in the genome of *B. napus* (Fig. 1I).

### Pollen and pollensomes reveal different lipid profiles under heat stress conditions

The lipid composition of pollen-derived extracts was analysed by GC-MS (Fig. 1J; Supplementary Dataset S1). Hexadecanoic (palmitic), 9-octadecenoic (oleic), and octadecanoic (stearic) acids were dominant in hydrated pollen (HP) and germinated pollen (GP) samples under Ctrl conditions. The same samples exposed to HS showed a strong increase in saturated fatty acids (SFAs), notably hexadecanoic acid (+57% in HP-HS and +19% in GP-HS) and pentadecanoic acid (+576% in HP-HS and +241% in GP-HS), and a decrease in long-chain SFAs, such as octadecanoic acid (−29% in HP-HS and −53% in GP-HS) and eicosanoic acid (arachidic acid; −20% in HP-HS and −55% in GP-HS). Interestingly, HS induced the reduction of docosanoic acid (behenic acid) to undetectable levels in both HP and GP, whereas 13-docosenoic acid (erucic acid), a Brassicaceae-specific very-long-chain fatty acid (VLCFA), increased moderately (+29% in HP-HS and +36% in GP-HS). The most dramatic changes occurred in PS, where triacontanoic acid, a VLCFA, represented 94.8% of total lipids in the Ctrl but disappeared completely under HS. Conversely, 9-octadecenoic acid, a monounsaturated fatty acid, increased from 5.2% in PS-Ctrl to 100% in PS-HS, thus becoming the sole detectable lipid in PS exposed to HS. No fatty acid was found in the EVF fraction under either treatment.

### Differential miRNA expression patterns are induced by heat stress

**Fig. 2.**
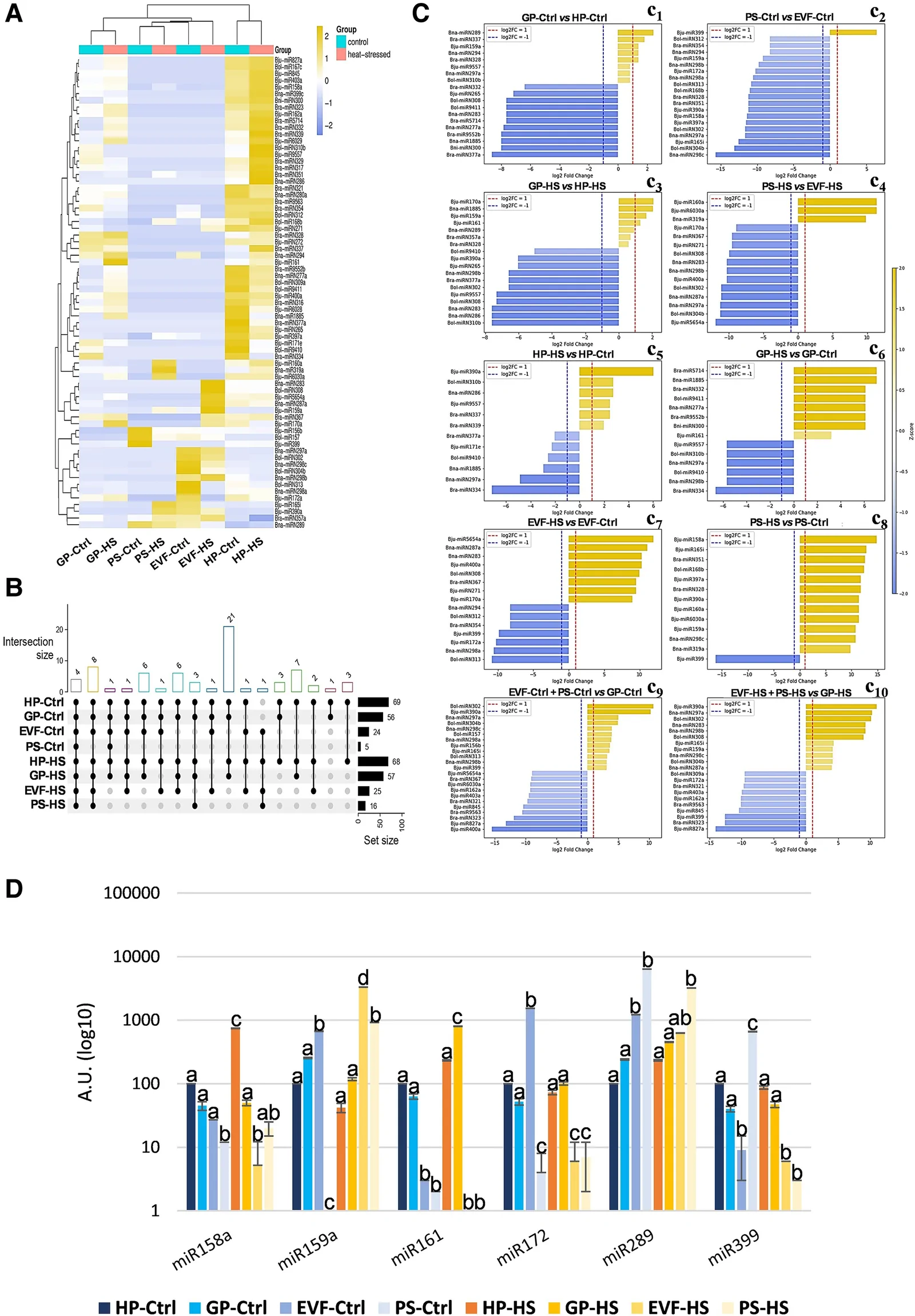
MiRNome analysis. (A) Hierarchically clustered heatmap depicting miRNA expression profiles between control (Ctrl) and heat stress (HS) treatments for germinated pollen (GP), the pollensome (PS) fraction, the extracellular vesicle-free (EVF) fraction, and hydrated pollen (HP). Differential expression is indicated according to the colour scale indicated in the key based on the *z*-score values measured for each miRNA in all samples (*P*-value<0.05). The clustering of control and heat-stressed groups is illustrated at the top of the figure. (B) UpSet plot illustrating the intersections of miRNA sets across the different samples. Each row below the graph represents a sample and the bars on the right indicate the total number of miRNAs detected per sample. Specific set intersections are indicated by the connected dots. The graph represents the number of miRNAs unique to each intersection, with the exact counts indicated. (C) Differentially expressed miRNAs identified in comparisons of different treatment and sample combinations, as indicated in each graph. Values of log_2_(fold-change) in expression are shown (based on the respective *z*-score values), as indicated in the colour scale to the right. A threshold of |*z*|≥1 was used to identify significantly differentially expressed miRNAs. (D) Relative abundance levels of selected miRNAs in the different samples as determined using RT-qPCR and expressed as arbitrary units (A.U.). The abundance is relative to that in the HP-Ctrl sample, the value of which was set as 100. Note the log scale. Data are means (±SD), *n*=3. Different letters within each miRNA indicate significant differences among the means as determined using ANOVA followed by Fisher’s LSD test (*P*<0.05).

Small-RNA sequencing identified a total of 70 distinct miRNAs across the HP, GP, EVF, PS sample types under Ctrl and HS conditions. Expression levels varied significantly according to the miRNA, pollen sample, and treatment (Supplementary Table S1). Bra-miRN357a exhibited the highest expression, with 804842 raw read counts in EVF-HS, followed by Bju-miR158a (224970 in HP-HS), and Bna-miRN289 (165432 in PS-Ctrl). Heatmap clustering analysis showed that Ctrl and HS plants were tightly grouped across the sample types (Fig. 2A), confirming the reproducibility and validity of the experiments. In addition, GP, PS, and EVF samples formed a cluster that was distinct from HP, indicating the existence of shared molecular features associated with pollen maturation and extracellular communication events. The relative impact of heat stress on each miRNA was further examined by calculating the percentage variation between HS and Ctrl conditions for each treatment–sample combination (Supplementary Table S2). This highlighted a selective response to heat stress, characterized by overall stability in the HP and GP samples and more dynamic modulations within the extracellular environment. Specifically, while the most-abundant miRNAs such as Bra-miRN357a remained remarkably stable, the EVF fraction showed the most dramatic shifts, with specific miRNAs such as Bju-miR159a and Bju-miR161 exhibiting increases of >800%, suggesting a key role for extracellular vesicles in the active stress response.

Comparative analysis revealed that HS induced the modulation of distinct miRNA patterns in the various samples. HP seemed to display the strongest response to the stress (35 miRNAs up-regulated and 35 down-regulated), while GP showed a milder response (36 miRNAs up-regulated and 27 down-regulated). In terms of the extracellular fractions, EVF showed a general repression of miRNAs (18 down-regulated compared with 14 up-regulated), whereas PS exhibited an opposite trend (13 up-regulated and 4 down-regulated). Bra-miR357 was the most abundant miRNA across all samples, regardless of the treatment. Specifically, the high expression of Bra-miR357 and Bju-miR158a in HP, and of Bra-miRN328 in GP, remained consistent between the Ctrl and HS conditions. Similarly, no shifts in the most-abundant miRNAs were observed in EVF samples. The only exception was in the PS fraction: while Bju-miR156b was among the most-expressed miRNAs under Ctrl conditions, Bna-miRN289 was the most-expressed following HS exposure.

Upset plots highlighted a structured scheme of overlap among the samples (Fig. 2B). HP and GP presented the largest sets of miRNAs (69 in Ctrl/68 in HS, and 56 in Ctrl/57 in HS, respectively), while EVF (24 in Ctrl/25 in HS) and PS (5 in Ctrl/16 in HS) the smallest ones, although presenting some miRNAs that were also detected in pollen. Four miRNAs (Bra-miRN357a, Bju-miR156b, Bna-miRN289, Bol-miR157) were consistently expressed across all samples. By contrast, three (Bra-miRN377a, Bju-miRN265, Bna-miRN286) were detectable exclusively in HP, while Bju-miR400a, Bju-miR6030a, and Bju-miR160a were found in HP and GP under both treatments and in PS-HS. Eight miRNAs (Bju-miR158a, Bra-miRN328, Bju-miR397a, Bra-miRN351, Bol-miR168b, Bna-miRN298c, Bju-miR165i, Bju-miR159a) were absent only in PS-Ctrl, whereas Bol-miRN302 was detected only in HP and EVF.

A total of 61 miRNAs showed differential expression between the Ctrl and HS treatments across the sample types (Fig. 2C). Comparing germinated and hydrated pollen (GP-Ctrl versus HP-Ctrl; Fig. 2C, c_1_), up-regulation of some miRNAs was found (e.g. Bju-miR337, Bju-miR289). Interestingly, the over-expression of Bju-miR159a and Bna-miRN289 also appeared to be shared in the same comparison exposed to HS (GP-HS versus HP-HS; Fig. 2C, c_3_), although additional HS-specific miRNAs were also detected, including Bju-miR170a and Bna-miR1885. It was notably that Bju-miR9557 and Bol-miRN310b, which were up-regulated in Ctrl conditions, became down-regulated under HS in these samples. Comparisons of PS-Ctrl versus EVF-Ctrl and PS-HS versus EVF-HS (Fig. 2, c_2_ and c_4_, respectively) showed that, overall, only four miRNAs were more abundant in PS samples compared to the respective EVF fractions, three of which were under HS (Bju-miR160, Bju-miR6030, Bna-miR319a) with only Bju-miR399 being up-regulated in PS-Ctrl with respect to EVF-Ctrl. Comparing samples of the same type (Fig. 2C, c_5-8_), PS exhibited the strongest contrast: in the presence of HS, only Bju-miR399 was down-regulated, suggesting a preferential stress-induced release into the vesicle-free medium (also according to the results in Fig. 2C, c_2_). GP released 12 miRNAs into the extracellular compartment in the Ctrl treatment and 11 under HS. Whilst some miRNAs were shared between these two sets (e.g. Bju-miR390a, Bna-miRN297a, Bol-miRN302), five were distinctive from EVF-Ctrl and PS-Ctrl (i.e. Bol-miR157, Bna-miRN298a, Bju-miR156b, Bol-miRN313, Bju-miR399) and four from the respective HS-fractions (i.e. Bna-miRN283, Bol-miRN308, Bju-miR159a, Bna-miRN287a). On the other hand, some miRNAs were retained by GP (e.g. Bju-miR845, Bra-miR9563, Bju-miR162a; Fig. 2C, c_9,10_).

To validate the small RNA-sequencing findings, six miRNAs (miR158a, miR159a, miR161, miR172, miR289, and miR399) were selected for RT-qPCR analysis based on their established roles in plant developmental plasticity and stress adaptation (Fig. 2D). Overall, the expression trends obtained were highly consistent with the sequencing-based quantification, providing independent confirmation of the heat-induced shifts in the miRNA landscape. In detail, the qPCR data confirmed a dramatic heat-induced accumulation of miR159a in the extracellular environment; specifically, relative to HP-Ctrl, the level of which was set as 100, the level in the EVF fraction increased to over 3000, while the PS fraction showed a level of ∼1000, which was a huge increase from its Ctrl level of close to zero. The results further validated the specific reduction of miR158a in the extracellular space (EVF and PS) compared to pollen grain samples (HP and GP) both under control and HS treatments. This trend was much more pronounced for miR161, the level of which dropped nearly to 1 related to HP-Ctrl. The analysis also indicated a significant reduction of miR172 in extracellular fractions subjected to HS, while in EVF-Ctrl its level increased to 1500. An even more pronounced trend was observed for miR399, which nearly vanished from the extracellular space upon HS treatment, showing a reduction of over 90%. In contrast, miR289 maintained consistently high levels across all fractions (ranging from 100–6000), independently of the application of HS.

### Predicted targets for miRNAs converge on developmental, hormonal, and stress-responsive pathways

Target prediction analysis for the 61 differentially expressed miRNAs revealed a wide list of proteins involved in various regulatory pathways (Table 1; Supplementary Dataset S2). A large fraction of targets belonged to families of developmental transcription factors, such as SQUAMOSA Promoter-Binding Proteins/SQUAMOSA Promoter-Binding Proteins-Like (SBP/SPL) (Bju-miR156b/Bol-miR157), APETALA2/Ethylene-Responsive Element Binding Factors (AP2/ERF) (Bju-miR172a), Homeodomain-Leucine Zipper Proteins (HD-ZIP) (Bju-miR165i), MYB transcription factors (Bju-miR159a/Bna-miR319a), and WRKY factors (WRKY domain family) (Bol-miRN302), indicating a central role for miRNAs in pollen development and floral reproductive organ morphogenesis. Another consistent category of miRNA targets was linked to hormone signalling, such as Bju-miR160a regulating Auxin Response Factor 17 (ARF17) and Bra-miRN367 acting on isopentenyl transferases, thus reinforcing the role of these short nucleic acids in cell growth and fate determination. Several targets encoded for cytoskeletal and motor proteins (e.g. myosin, villin, and actin-binding domains for Bju-miR9557, Bol-miR9411, and Bol-miRN312, respectively), suggesting regulation of structural rearrangements essential for endomembrane trafficking and pollen-tube development. Among the predictions, several transcripts were also potentially associated with plant extracellular vesicles: these included membrane transporters, such as ABC transporter G family members (Bju-miRN271), the sucrose-proton symporter SUC1 (Bju-miR845), and members of the NRT1/PTR family (Bol-miRN308), all localized to the plasma membrane and frequently transported through vesicular pathways. Lipid- and membrane-modifying enzymes also appeared as targets, including GPI7 (Bra-miRN377a), ATS1 (Bol-miR9411), and PDAT (Bra-miRN357a), indicating that miRNA regulation might influence processes relevant to vesicle membrane composition and biogenesis. Additionally, Bju-miR158a (TRIACYLGLYCEROL LIPASE/TRIGLYCERIDE LIPASE) and Bju-miR399 (ATBZIP TRANSCRIPTION FACTOR-RELATED) were predicted to regulate lipid metabolism and phosphate homeostasis, respectively, both of which are crucial for membrane remodelling and metabolic adaptation under stress. The association of Bni-miRN300 with TOLL/INTERLEUKIN-1 receptor homology (TIR) domain-containing protein and of Bra-miRN354 with trafficking protein particle complex subunit 9 could suggest regulatory roles for these modules in immune signalling and intracellular vesicle movements. Bju-miR390a was predicted to regulate flavonoid 3′-monooxygenase/hydroxylase, while Bra-miRN334 was predicted to regulate the flavonol 3-*O*-glucosyltransferase, indicating their possible involvement in the modulation of flavonoid biosynthesis during pollen development and in stress acclimation mechanisms. Finally, disease-resistance proteins (e.g. Nucleotide-binding Leucine-rich Repeat receptors, NLRs; Leucine-rich Repeat proteins, LRRs) and ubiquitin ligases, targeted by Bju-miR390a, Bju-miR5654a, and Bju-miR6030a, highlighted the activation of pathways related to stress response, protein turnover, and reproductive regulation.

**Table 1.** Summary of target prediction analysis for the differentially expressed miRNAs induced by heat stress across the different pollen fractions of *Brassica napus*

Query		Target		
	**Exp value**	**Accession**	**Arabidopsis ortholog**	**Description**
Bju-miR156b	0.0	Bol023958	AT1G69170	SQUAMOSA PROMOTER BINDING PROTEIN (SBP)-DOMAIN TRANSCRIPTION FACTOR 6
	1.0	Bol028594	AT1G69170	SQUAMOSA PROMOTER-BINDING-LIKE PROTEIN 15-RELATED
Bra-miRN367	0.0	Bol037304	AT3G23630	ADENYLATE DIMETHYLALLYLTRANSFERASE (AMP-DEPENDENT)/ISOPENTENYLTRANSFERASE
	0.0	Bol014450	AT3G63110	ADENYLATE ISOPENTENYLTRANSFERASE 3, CHLOROPLASTIC
	0.0	Bol007141	AT5G19040	ADENYLATE ISOPENTENYLTRANSFERASE 3, CHLOROPLASTIC
	0.0	Bol028718	AT3G23630	ADENYLATE DIMETHYLALLYLTRANSFERASE (AMP-DEPENDENT)/ISOPENTENYLTRANSFERASE
	0.5	Bol044624	AT3G63110	ISOPENTENYLTRANSFERASE 3
	0.5	Bol038222	AT1G16410	CYTOCHROME P450, FAMILY 79, SUBFAMILY F, POLYPEPTIDE 1 (CYP79F1)
			AT1G16400	CYTOCHROME P450, FAMILY 79, SUBFAMILY F, POLYPEPTIDE 2 (CYP79F2)
	1.0	Bol014680	AT5G19040	ISOPENTENYLTRANSFERASE 5
Bju-miR165i	0.5	Bol035052	AT1G52150	HOMEOBOX-LEUCINE ZIPPER PROTEIN ATHB-15
	0.5	Bol019484	AT1G52150	HOMEOBOX-LEUCINE ZIPPER PROTEIN ATHB-15
	1.0	Bol037848	AT2G34710	HOMEOBOX-LEUCINE ZIPPER PROTEIN ATHB-14
	1.0	Bol022567	AT1G30490	HOMEOBOX-LEUCINE ZIPPER PROTEIN ATHB-14-RELATED
	1.0	Bol017514	AT4G32880	HOMEOBOX-LEUCINE ZIPPER PROTEIN ATHB-8
	1.0	Bol017856	AT4G32880	HOMEOBOX-LEUCINE ZIPPER PROTEIN ATHB-8
	1.0	Bol035990	AT5G60690	HOMEOBOX DOMAIN (HOMEOBOX)//START DOMAIN (START)//MEKHLA DOMAIN (MEKHLA)
Bju-miR172a	0.5	Bol044334	AT3G54990	AP2-LIKE ETHYLENE RESPONSIVE TRANSCRIPTION FACTOR SMZ-RELATED
	1.0	Bol008932	AT5G60120	AP2-LIKE ETHYLENE-RESPONSIVE TRANSCRIPTION FACTOR SMZ-RELATED
	1.0	Bol017251	AT5G60120	AP2-LIKE ETHYLENE-RESPONSIVE TRANSCRIPTION FACTOR SMZ-RELATED
	1.0	Bol028934	AT4G36920	FLORAL HOMEOTIC PROTEIN APETALA 2
	1.0	Bol018627	AT4G36920	FLORAL HOMEOTIC PROTEIN APETALA 2
	1.0	Bol032944	AT2G28550	ETHYLENE-RESPONSIVE TRANSCRIPTION FACTOR RAP2-7
	1.0	Bol008371	AT2G28550	ETHYLENE-RESPONSIVE TRANSCRIPTION FACTOR RAP2-7
Bni-miRN300	0.5	Bol010192	AT5G11250	LEUCINE-RICH REPEAT-CONTAINING PROTEIN//DISEASE RESISTANCE RPP5 LIKE PROTEIN-RELATED
	1.0	Bol010187	AT3G04220	TOLL/INTERLEUKIN-1 RECEPTOR HOMOLOGY (TIR) DOMAIN
Bju-miR159a	1.0	Bol008198	AT2G32460	MYB FAMILY TRANSCRIPTION FACTOR
Bju-miR160a	1.0	Bol027532	AT1G77850	AUXIN RESPONSE FACTOR 17
Bju-miR161	1.0	Bol031408	AT1G12300	RFL2
Bju-miR397a	1.0	Bol017577	AT2G29130	LACCASE-2
Bju-miR400a	1.0	Bol029868	AT4G19440	TETRATRICOPEPTIDE REPEAT (TPR)-LIKE SUPERFAMILY PROTEIN
Bna-miR1885	1.0	Bol007144	AT5G18370	LEUCINE-RICH REPEAT DOMAIN (NLR) RECEPTOR (DSC2)
Bol-miRN302	1.0	Bol018552	AT4G39410	WRKY TRANSCRIPTION FACTOR 13-RELATED
Bju-miR162a	1.5	Bol016383	AT5G25757	TRANSLATION INITIATION FACTOR 3 SUBUNIT L (EIF3L)
Bju-miR171e	1.5	Bol041519	AT4G36710	SCARECROW-LIKE PROTEIN 15
Bju-miR399	1.5	Bol029939	AT4G45900	ATBZIP TRANSCRIPTION FACTOR-RELATED
Bju-miR5654a	1.5	Bol031407	AT1G12620	PPR REPEAT FAMILY (PPR_2)
	1.5	Bol045532	AT1G12620	PPR REPEAT FAMILY (PPR_2)
	1.5	Bol031494	AT1G12620	PPR REPEAT FAMILY (PPR_2)
	1.5	Bol031388	AT1G12300	PPR REPEAT FAMILY (PPR_2)//PENTATRICOPEPTIDE REPEAT DOMAIN (PPR_3)
	1.5	Bol035842	AT1G12620	PPR REPEAT FAMILY (PPR_2)
	1.5	Bol031351	AT1G12300	PPR REPEAT FAMILY (PPR_2)//PENTATRICOPEPTIDE REPEAT DOMAIN (PPR_3)
	1.5	Bol029121	AT1G12620	PPR REPEAT FAMILY (PPR_2)
	1.5	Bol031370	AT1G12775	PPR REPEAT FAMILY (PPR_2)//PENTATRICOPEPTIDE REPEAT DOMAIN (PPR_3)
			AT1G12620	PPR REPEAT FAMILY (PPR_2)
			AT1G12300	PPR REPEAT FAMILY (PPR_2)//PENTATRICOPEPTIDE REPEAT DOMAIN (PPR_3)
	1.5	Bol031371	AT1G12775	PPR REPEAT FAMILY (PPR_2)//PENTATRICOPEPTIDE REPEAT DOMAIN (PPR_3)
			AT1G12620	PPR REPEAT FAMILY (PPR_2)
			AT1G12300	PPR REPEAT FAMILY (PPR_2)//PENTATRICOPEPTIDE REPEAT DOMAIN (PPR_3)
	1.5	Bol031387	AT1G12775	PPR REPEAT FAMILY (PPR_2)//PENTATRICOPEPTIDE REPEAT DOMAIN (PPR_3)
			AT1G12620	PPR REPEAT FAMILY (PPR_2)
			AT1G12300	PPR REPEAT FAMILY (PPR_2)//PENTATRICOPEPTIDE REPEAT DOMAIN (PPR_3)
	1.5	Bol031450	AT1G12775	PPR REPEAT FAMILY (PPR_2)//PENTATRICOPEPTIDE REPEAT DOMAIN (PPR_3)
			AT1G12620	PPR REPEAT FAMILY (PPR_2)
			AT1G12300	PPR REPEAT FAMILY (PPR_2)//PENTATRICOPEPTIDE REPEAT DOMAIN (PPR_3)
	1.5	Bol045166	AT1G12280	LEUCINE-RICH REPEAT-CONTAINING PROTEIN//DISEASE RESISTANCE PROTEIN RFL1-RELATED
Bna-miR319a	1.5	Bol008198	AT2G32460	MYB FAMILY TRANSCRIPTION FACTOR
	1.5	Bol027782	AT2G26960	MYB PROTO-ONCOGENE PROTEIN, PLANT (MYBP)
	1.5	Bol032889	AT2G26960	MYB PROTO-ONCOGENE PROTEIN, PLANT (MYBP)
	1.5	Bol023199	AT3G11440	MYB-LIKE DNA-BINDING PROTEIN MYB//MYB TRANSCRIPTION FACTOR
	1.5	Bol005908	AT3G11440	MYB-LIKE DNA-BINDING PROTEIN MYB//MYB TRANSCRIPTION FACTOR
Bol-miR157	1.5	Bol016006	AT1G27370	SQUAMOSA PROMOTER-BINDING-LIKE PROTEIN 10-RELATED
	1.5	Bol027130	AT5G43270	SQUAMOSA PROMOTER-BINDING-LIKE PROTEIN 10-RELATED
	1.5	Bol011022	AT3G57920	SQUAMOSA PROMOTER-BINDING-LIKE PROTEIN 15-RELATED
	1.5	Bol007052	AT3G57920	SQUAMOSA PROMOTER-BINDING-LIKE PROTEIN 15-RELATED
	1.5	Bol004847	AT2G42200	SBP domain (SBP)
	1.5	Bol041769	AT1G27360	SQUAMOSA PROMOTER-BINDING-LIKE PROTEIN 10-RELATED
			AT1G27370	SQUAMOSA PROMOTER-BINDING-LIKE PROTEIN 10-RELATED
Bol-miRN308	1.5	Bol004377	AT1G27040	OLIGOPEPTIDE TRANSPORTER-RELATED//PROTEIN NRT1/PTR FAMILY 4.5-RELATED
Bra-miRN323	1.5	Bol024303	AT4G21440	MYB-LIKE DNA-BINDING PROTEIN MYB//MYB TRANSCRIPTION FACTOR
Bju-miR403a	2.0	Bol031459	AT1G13940	T-BOX TRANSCRIPTION FACTOR, PUTATIVE (DUF863)
Bju-miRN265	2.0	Bol038221	AT1G16380	CATION/H (+) ANTIPORTER 1-RELATED (ATCHX1)
Bju-miRN271	2.0	Bol025127	AT3G53510	ATP-BINDING CASSETTE TRANSPORTER//ABC TRANSPORTER G FAMILY MEMBER 1-RELATED
	2.0	Bol017079	AT5G23110	SACSIN
Bna-miRN286	2.0	Bol026888	AT1G19770	PURINE PERMEASE 11-RELATED (ATPUP14)
Bna-miRN297a	2.0	Bol043713	AT5G09970	CYTOCHROME P450 78A7
	2.0	Bol012396	AT4G11440	MITOCHONDRIAL SUBSTRATE CARRIER FAMILY PROTEIN
Bol-miRN309a	2.0	Bol022958	AT3G17090	PROTEIN PHOSPHATASE 2C 42-RELATED
Bol-miRN310b	2.0	Bol009417	NA	NA
Bra-miR5714	2.0	Bol034905	AT3G30820	ATHILA ORF-1 family (ATHILA)
Bra-miR9552b	2.0	Bol026297	AT1G73050	MANDELONITRILE LYASE (E4.1.2.10)
Bra-miRN321	2.0	Bol015973	NA	NA
Bra-miRN339	2.0	Bol044810	AT2G20635	MITOTIC CHECKPOINT SERINE/THREONINE-PROTEIN KINASE BUB1
Bra-miRN354	2.0	Bol043608	AT5G11040	TRAFFICKING PROTEIN PARTICLE COMPLEX SUBUNIT 9
	2.0	Bol036905	NA	NA
Bju-miR158a	2.5	Bol044710	AT2G23540	TRIACYLGLYCEROL LIPASE/TRIGLYCERIDE LIPASE
Bju-miR170a	2.5	Bol041519	AT4G36710	SCARECROW-LIKE PROTEIN 15
Bju-miR390a	2.5	Bol041146	AT1G07650	LEUCINE RICH REPEAT (LRR_1)//PROTEIN TYROSINE KINASE (PKINASE_TYR)//DI-GLUCOSE BINDING WITHIN ENDOPLASMIC RETICULUM (MALECTIN)//LEUCINE RICH REPEAT (LRR_8)
	2.5	Bol040710	AT1G01190	FLAVONOID 3′-MONOOXYGENASE/FLAVONOID 3′-HYDROXYLASE
	2.5	Bol029211	AT1G14510	ALFIN-LIKE 7
	2.5	Bol038049	AT1G14510	ALFIN-LIKE 7
Bju-miR6030a	2.5	Bol045166	AT1G12280	LEUCINE-RICH REPEAT-CONTAINING PROTEIN//DISEASE RESISTANCE PROTEIN RFL1-RELATED
	2.5	Bol009691	AT1G21790	PROTEIN K12H6.6, ISOFORM B-RELATED
Bju-miR845	2.5	Bol034930	AT1G71880	SUCROSE-PROTON SYMPORTER 1
Bju-miR9557	2.5	Bol036954	AT2G34730	MYOSIN HEAVY CHAIN-RELATED
Bna-miRN287a	2.5	Bol032785	AT3G48560	ACETOLACTATE SYNTHASE I/II/III LARGE SUBUNIT (E2.2.1.6L, ILVB, ILVG, ILVI)
Bna-miRN294	2.5	Bol016064	AT4G38640	CTL TRANSPORTER//PLASMA-MEMBRANE CHOLINE TRANSPORTER FAMILY PROTEIN
Bna-miRN298a	2.5	Bol022704	AT2G19270	MITOTIC CHECKPOINT PROTEIN PRCC
	2.5	Bol008854	AT5G07572	HYPOTHETICAL PROTEIN
Bna-miRN298b	2.5	Bol040221	AT5G37510	NADH DEHYDROGENASE (UBIQUINONE) FE-S PROTEIN 1 (NDUFS1)
Bol-miRN304b	2.5	Bol010729	AT3G01730	MUTANTS EXHIBIT SHORTER ROOT HAIRS UNDER PHOSPHATE-DEFICIENT CONDITIONS
	2.5	Bol040154	AT5G38560	PROLINE-RICH RECEPTOR-LIKE PROTEIN KINASE PERK8
Bol-miRN313	2.5	Bol009683	AT3G49050	ALPHA/BETA-HYDROLASES SUPERFAMILY PROTEIN
	2.5	Bol028813	AT4G39350	CELLULOSE SYNTHASE (UDP-FORMING)/UDP-GLUCOSE-CELLULOSE GLUCOSYLTRANSFERASE
Bra-miRN332	2.5	Bol031601	NA	NA
	2.5	Bol033354	AT2G27040	EUKARYOTIC TRANSLATION INITIATION FACTOR 2C//PROTEIN ARGONAUTE 4-RELATED
Bra-miRN334	2.5	Bol031420	AT1G13120	NUCLEOPORIN GLE1 (GLE1)
	2.5	Bol042193	AT4G24830	ARGININOSUCCINATE SYNTHASE/CITRULLINE–ASPARTATE LIGASE
	2.5	Bol035958	NA	TRANSPOSASE FAMILY TNP2 (TRANSPOSASE_21)//TRANSPOSASE-ASSOCIATED DOMAIN (TRANSPOS_ASSOC)
	2.5	Bol031911	AT2G15490	FLAVONOL 3-O-GLUCOSYLTRANSFERASE/UDP-GLUCOSE FLAVONOL 3-O-GLUCOSYLTRANSFERASE
			AT2G15480	FLAVONOL 3-O-GLUCOSYLTRANSFERASE/UDP-GLUCOSE FLAVONOL 3-O-GLUCOSYLTRANSFERASE
	2.5	Bol042616	AT2G15490	FLAVONOL 3-O-GLUCOSYLTRANSFERASE/UDP-GLUCOSE FLAVONOL 3-O-GLUCOSYLTRANSFERASE
			AT2G15480	FLAVONOL 3-O-GLUCOSYLTRANSFERASE/UDP-GLUCOSE FLAVONOL 3-O-GLUCOSYLTRANSFERASE
Bna-miRN283	3.0	Bol006590	AT5G44400	BERBERINE BRIDGE ENZYME-RELATED (ATBBE26)
	3.0	Bol037762	AT2G35910	RING-H2 FINGER PROTEIN ATL70
	3.0	Bol038803	AT5G20550	2-OXOGLUTARATE (2OG) AND FE(II)-DEPENDENT OXYGENASE SUPERFAMILY PROTEIN
Bna-miRN289	3.0	Bol016881	AT5G58610	ACYL-COA N-ACYLTRANSFERASE WITH RING/FYVE/PHD-TYPE ZINC FINGER DOMAIN-RELATED
	3.0	Bol034615	AT4G23590	TYROSINE TRANSAMINASE FAMILY PROTEIN
Bna-miRN298c	3.0	Bol045818	AT2G22900	GLYCOSYLTRANSFERASE 6-RELATED
Bol-miR168b	3.0	Bol043177	AT1G48410	EUKARYOTIC TRANSLATION INITIATION FACTOR 2C//SUBFAMILY NOT NAMED
	3.0	Bol023381	AT1G07360	PRE-MRNA-SPLICING FACTOR RBM22/SLT11 (RBM22, SLT11)
Bol-miR9410	3.0	Bol025993	AT5G58210	EXO-ALPHA-SIALIDASE/SIALIDASE
Bra-miRN328	3.0	Bol011560	AT1G04880	HMG BD15
Bra-miRN337	3.0	Bol010001	AT5G04180	CARBONIC ANHYDRASE//ALPHA CARBONIC ANHYDRASE 3
	3.0	Bol006221	AT5G55600	BAH DOMAIN (BAH)//AGENET DOMAIN (AGENET)
Bra-miRN351	3.0	Bol025720	AT3G14810	MECHANOSENSITIVE ION CHANNEL PROTEIN 4-RELATED
	3.0	Bol043761	AT5G09220	AMINO ACID TRANSPORTER//AMINO ACID PERMEASE 2
	3.0	Bol025374	AT2G39940	CORONATINE-INSENSITIVE PROTEIN 1 (COI-1)
	3.0	Bol027480	AT1G17100	HAEM-BINDING PROTEIN 1 (ATHBP1)
	3.0	Bol007924	AT1G01690	PUTATIVE RECOMBINATION INITIATION DEFECTS 3
Bra-miRN357a	3.0	Bol018408	AT4G02570	CULLIN 1 (CUL1)
	3.0	Bol033628	AT4G29960	EBS7
	3.0	Bol024089	AT3G47940	DNAJ HOMOLOG SUBFAMILY C MEMBER//DNAJ HEAT SHOCK PROTEIN-RELATED
	3.0	Bol018368	AT1G04010	PHOSPHOLIPID:DIACYLGLYCEROL ACYLTRANSFERASE/PDAT//1-ACYLGLYCEROPHOSPHOCHOLINE O-ACYLTRANSFERASE/LYSOLECITHIN ACYLTRANSFERASE
	3.0	Bol010029	AT5G03790	HOMEODOMAIN LEUCINE ZIPPER CLASS I (HD-ZIP I)
	3.0	Bol026354	AT1G72300	RECEPTOR LIKE PROTEIN 3-RELATED
Bra-miRN377a	3.0	Bol045826	AT2G22530	ETHANOLAMINEPHOSPHOTRANSFERASE (GPI7)
	3.0	Bol006934	AT2G41640	GLYCOSYLTRANSFERASE//SUBFAMILY NOT NAMED
	3.0	Bol027051	AT4G14050	PPR REPEAT (PPR)//PPR REPEAT FAMILY (PPR_2)//DYW FAMILY OF NUCLEIC ACID DEAMINASES (DYW_DEAMINASE)
	3.0	Bol012184	AT5G57830	MYOB12
	3.0	Bol007988	AT3G50050	ASPARTYL PROTEASES//SUBFAMILY NOT NAMED
Bju-miR827a	3.5	Bol016150	AT1G71220	UDP-GLUCOSE:GLYCOPROTEIN GLUCOSYLTRANSFERASE (HUGT)
	3.5	Bol042678	AT1G49150	TRANSMEMBRANE PROTEIN
	3.5	Bol014470	AT3G63340	PROTEIN PHOSPHATASE 2C//PROTEIN PHOSPHATASE 2C 50-RELATED
Bna-miRN277a	3.5	Bol010214	NA	ULP1 PROTEASE FAMILY, C-TERMINAL CATALYTIC DOMAIN (PEPTIDASE_C48)
	3.5	Bol044284	NA	ULP1 PEPTIDASE/ULP1 PROTEASE
	3.5	Bol030633	AT4G02930	TRANSLATION FACTOR//SUBFAMILY NOT NAMED
	3.5	Bol013958	AT3G24560	TRNA (ILE)-LYSIDINE SYNTHETASE/ISOLEUCINE-SPECIFIC TRANSFER RIBONUCLEATE LYSIDINE SYNTHETASE
Bol-miR9411	3.5	Bol025680	AT4G10640	PROTEIN IQ-DOMAIN 17-RELATED
	3.5	Bol008111	AT3G06600	SINE3
	3.5	Bol017740	AT2G37950	MEMBRANE ASSOCIATED RING FINGER//SUBFAMILY NOT NAMED
	3.5	Bol018711	AT4G35470	LEUCINE-RICH REPEAT-CONTAINING PROTEIN//SUBFAMILY NOT NAMED (PIRL4)
	3.5	Bol022253	AT1G32200	GLYCEROL-3-PHOSPHATE O-ACYLTRANSFERASE (ATS1)
	3.5	Bol020538	AT2G41740	VILLIN-2-RELATED
	3.5	Bol006948	AT2G41740	VILLIN-2-RELATED
Bol-miRN312	3.5	Bol035545	AT3G13672	SEVEN IN ABSENTIA HOMOLOG//E3 UBIQUITIN-PROTEIN LIGASE
	3.5	Bol030741	AT1G18750	MADS-BOX PROTEIN (AGL65)
	3.5	Bol032324	AT5G48460	FIMBRIN/PLASTIN//ACTIN BINDING CALPONIN HOMOLOGY DOMAIN-CONTAINING PROTEIN-RELATED
	3.5	Bol017248	AT5G60150	HYPOTHETICAL PROTEIN
Bra-miR9563	3.5	Bol023399	AT1G07630	PROTEIN PHOSPHATASE 2C 36-RELATED (PLL5)
	3.5	Bol041143	AT1G07630	PROTEIN PHOSPHATASE 2C//PROTEIN PHOSPHATASE 2C 36-RELATED
	3.5	Bol035831	AT5G20280	SUCROSE-PHOSPHATE SYNTHASE 1-RELATED
	3.5	Bol024410	AT4G18790	NATURAL RESISTANCE-ASSOCIATED MACROPHAGE PROTEIN (SLC11A, NRAMP)

For each investigated miRNA (Query), the putative target mRNAs with the lowest expectation (Exp) value are listed as accession number and as the *Arabidopsis thaliana* ortholog ID (using the ‘one-to-one’, ‘one-to-many’, ‘many-to-many’, ‘many-to-one’ functions by BioMart on Phytozome or by the BRAD database annotation tool BLASTX). When available, targets with Exp≤1 were prioritized. In addition, a description of each target is provided (NA, not annotated). The complete output of the bioinformatics analysis is provided in Supplementary Dataset S2. Abbreviations: Bju, *Brassica juncea*; Bna, *B. napus*; Bni, *B. nigra*; Bol, *B. oleracea*; Bra, *B. rapa*.

### MiRNA activity orchestrates reproductive development and vesicle dynamics

**Fig. 3.**
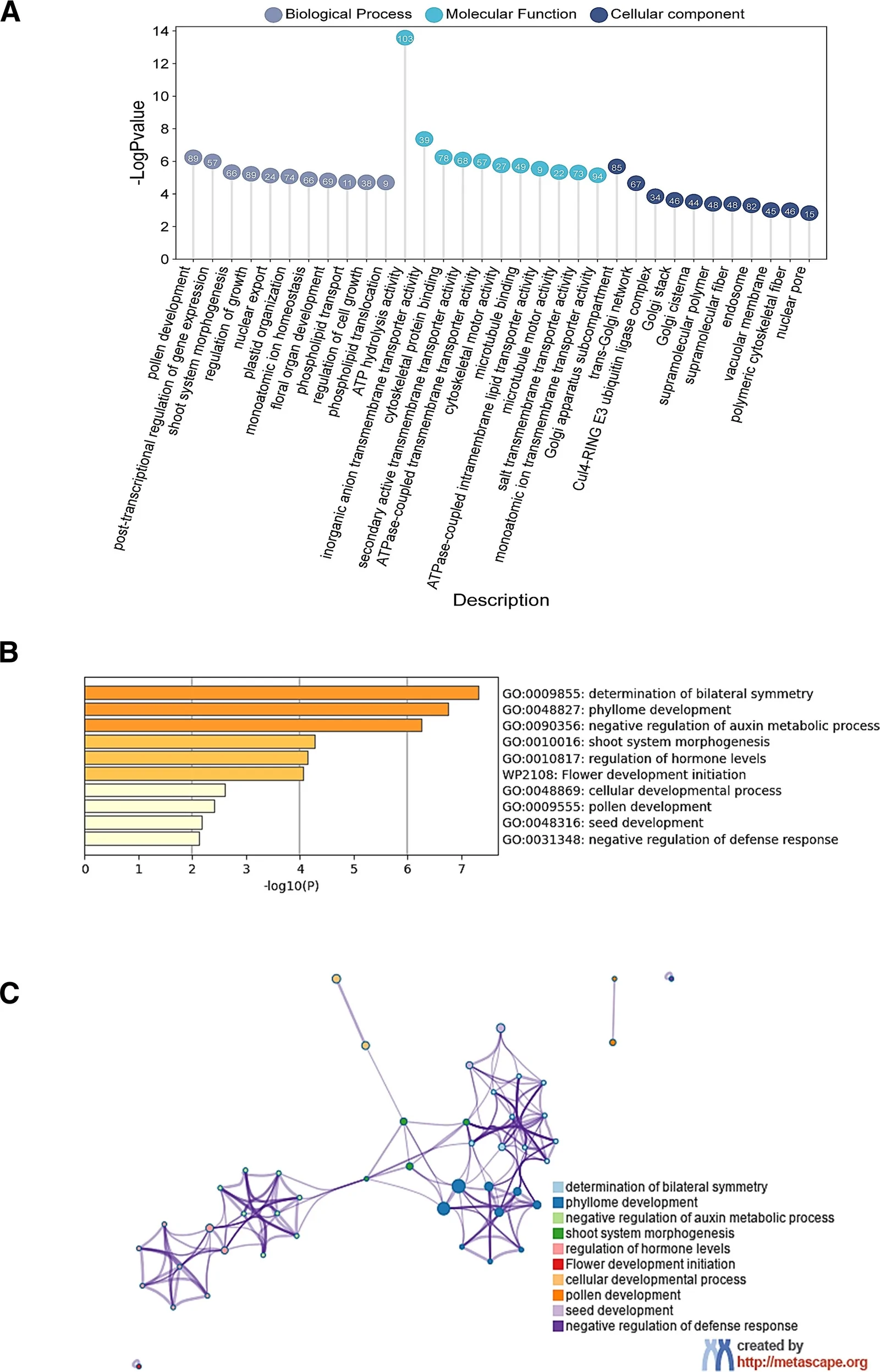
Enrichment analysis of the targets of differentially expressed miRNAs across the different pollen fractions of *Brassica napus*. A total of 61 miRNAs were found to be significantly differentially expressed across the samples, and their predicted targets are listed in Table 1. (A) Enrichment analysis based on clusterProfiler. The GO terms are grouped by category as indicated, and the number of genes associated with each term is indicated. (B) Enrichment analysis performed by Metascape. The GO terms are ranked by statistical significance and the different levels are further highlighted by the different shading. (C) Network visualization performed by Metascape. The network visualizes a subset of representative GO terms indicated as circles, the size of which is proportional to the number of input genes associated with that term. Node colour indicates the cluster identity, grouping functionally related terms. Edges connect terms with a similarity score >0.3, while the edge thickness reflects the degree of similarity. The most statistically significant term within a cluster was chosen as the one representing the cluster.

Functional enrichment analysis of the miRNA targets using clusterProfiler revealed over-representation of reproductive and cellular processes (Fig. 3A). Among Biological Processes, pollen development was the most significantly enriched term (−log*P*=6.25, 89 genes), together with floral organ development (−log*P*=4.83, 69 genes), shoot system morphogenesis (−log*P*=5.34, 66 genes), and post-transcriptional regulation of gene expression (−log*P*=5.99, 57 genes), indicating a central role for miRNAs in reproductive organogenesis and stress-induced gene reprogramming. Additionally, there was enrichment in phospholipid transport (−log*P*=4.74, 11 genes), confirming the existence of lipid remodelling already observed in the lipidomic analyses. Within Molecular Function, ATP hydrolysis activity dominated (−log*P*=13.59, 103 genes), along with transporter- and cytoskeleton-related categories. Specifically, inorganic anion transmembrane transporter activity (−log*P*=7.37, 39 genes), cytoskeletal protein binding (−log*P*=6.26, 78 genes), and ATPase-coupled intramembrane lipid transporter activity (−log*P*=5.55, nine genes) highlighted potential roles for the detected miRNAs in coordinating both ion/lipid fluxes and structural rearrangements. Enriched terms within Cellular Components were mainly linked to vesicle formation and trafficking, including Golgi apparatus subcompartment (−log*P*=5.69, 85 genes), *trans*-Golgi network (−log*P*=4.65, 67 genes), and endosome (−logP=3.29, 82 genes). Categories such as supramolecular fiber and supramolecular (e.g. cytoskeletal) polymer (both −log*P*=3.39, 48 genes) suggested a link between vesicle biogenesis and cytoskeletal dynamics. Notably, the category vacuolar membrane (−log*P*=3.01, 45 genes) was also present, further corroborating the involvement of miRNAs in lipid-related compartment remodelling. Complementary analysis using Metascape confirmed the effect of the miRNA set with the presence of genes involved in developmental and morphogenetic processes (Fig. 3B). The most significantly enriched categories included determination of bilateral symmetry determination, phyllome development, and negative regulation of auxin metabolic process, followed by shoot system morphogenesis and regulation of hormone levels. Processes directly associated with reproduction were also found, including flower development initiation, cellular developmental process, and pollen development. Finally, seed development (GO:0048316) and negative regulation of defense response were detected, although at a lower statistical significance. Consistent with this, network analysis revealed tightly connected clusters corresponding to these functional categories (Fig. 3C). Developmental processes including determination of bilateral symmetry, phyllome development, shoot system morphogenesis, and regulation of hormone levels formed the major cluster, while a second group was centred on reproductive processes, including flower development initiation and pollen development. The strong interconnection among these categories underscored their coordinated regulation and the role of the miRNA set in the reproductive transition. Stress- and defense-related terms were less interconnected but still present, suggesting the activation of functions related to acclimation and environmental adaptation.

## Discussion

Heat stress represents one of the major challenges for agricultural productivity, especially under current scenarios of rapid climate change. Elevated temperatures negatively affect multiple physiological and reproductive processes in plants, including floral organ development and fertilization, ultimately reducing seed set (Young *et al*., 2004; Rosenberger *et al*., 2024). In this context, understanding how heat stress influences both pollen biology and the release of pollensomes (PS) is crucial to determining the molecular basis of heat-induced male sterility. Our present study aimed at addressing this knowledge gap by investigating the effects of a sustained heat-stress (HS) treatment on pollen performance and PS in the *B. napus* cultivar ‘Phoenix CL’. Compared with controls, HS plants showed a marked reduction in pollen productivity, in agreement with previous evidence describing decreased floral production and increased abortion under elevated temperatures during the flowering stage (Magno Massuia de Almeida *et al*., 2022). However, the pollen produced by HS plants showed neither morphological abnormalities nor reduced viability. While this lack of impact on pollen viability might seem surprising, it is important to note that the effects of heat stress can depend on several factors: the maximum temperature reached, the duration of the stress, the phenological stage at which it occurs, and the cultivar studied. Indeed, whilst a study by Lohani *et al*. (2021) on *B. napus* has reported a decrease in pollen viability when imposing a high heat stress for a short duration (40 °C for 30 min), another study by Young *et al*. (2004) employing a protocol comparable to the HS treatment we used here has not found any significant decrease in either viability or germinability of *B. napus* pollen. In our study, HS instead caused a marked decrease in pollen germinability (Fig. 1A). Taken together, these results suggest that prolonged heat stress might enable plants to activate defense mechanisms aimed at protecting pollen viability. Since each study used a different cultivar, they also indicate that this could be a factor in the response, highlighting the importance of cultivar selection in the agricultural adaptation to climate change.

Nanoparticle tracking analysis revealed their presence in the PS fraction of both the HS and Ctrl samples (Fig. 1B–G), with estimated diameters comparable to those previously published for PS and for plant extracellular vesicles (EVs) in general. Western blotting confirmed the presence of positive markers of PS and excluded cellular contamination in this fraction (Fig. 1I; Kong *et al*., 2014; Suanno *et al*., 2023). Thus, the morphological and molecular characterization of the PS fraction carried out in this study is consistent with the current literature, indicating that *B. napus* pollen externalizes EVs during germination. While the HS treatment did not consistently affect PS size, it significantly increased the quantity of PS secreted during germination, possibly as a compensation for the decrease in germinability, thus suggesting first that high temperature might modulate the cellular machinery responsible for vesicle formation and secretion, and second that PS secretion could be involved in the fertilization process. The lower germination rate of the HS pollen might have been a consequence of the altered lipidic composition of its membranes (Fig. 1J). Fatty acids are known to play a dual role in pollen biology, acting both as structural components of membranes and as key modulators during fertilization events. The lipid-rich pollen coat, particularly abundant in Brassicaceae, is dominated by saturated acyl esters, such as palmitic and stearic acids, and contributes to both physical protection and pollen–stigma communication (Wolters-Arts *et al*., 1998; Dickinson *et al*., 2000; Ischebeck, 2016). Moreover, the rapid growth of pollen tubes relies on sustained membrane biogenesis, supported by *de novo* fatty acid synthesis and mobilization of lipid droplets (Ischebeck, 2016). VLCFAs, alkanes, and esters are particularly dominant in Brassicaceae pollen and seem to be required for its fertility and stigma recognition (Piffanelli *et al*., 1998; Fiebig *et al*., 2000).

In our samples, HS triggered marked lipidomic remodelling in hydrated and germinated pollen, as well as in pollen-derived extracellular vesicles. The observed increase in saturated fatty acids and the reduction in long-chain and very-long-chain species suggest a balancing mechanism aimed at maintaining membrane functionality under elevated temperatures, in line with established models of stress-induced lipid adjustment (Larkindale and Huang, 2004; Narayanan *et al*., 2016). Notably, the loss of triacontanoic acid, a VLCFA, from pollensomes, coupled with the near-complete replacement by oleic acid (Fig. 1J), indicated a profound reorganization of vesicle membrane composition. Given the structural role of VLCFAs in stabilizing hydrophobic barriers (Kunst and Samuels, 2009; Batsale *et al*., 2021), this shift might influence vesicle stability and fusion capacity. Conversely, the accumulation of oleic acid, a lipid associated with membrane fluidization and stress resilience, could reflect an adaptive response (Upchurch, 2008; Lim *et al*., 2017). To our knowledge, such an extreme heat stress-driven lipid switch in extracellular vesicles has not previously been reported and it might represent an as yet undefined strategy of pollen–pistil communication or stress adaptation in rapeseed mediated by the lipid cargo. The moderate increase in erucic acid is consistent with temperature-dependent remodelling of Brassicaceae lipid pools (Sharafi *et al*., 2015), suggesting a key role for this fatty acid. Overall, all the detected lipidomic changes suggest that HS altered not only the intracellular lipid homeostasis but also the composition and possibly the function of extracellular vesicles, with potential consequences for their trafficking and involvement in pollen–pistil communication (Pleskot *et al*., 2016; Jiang *et al*., 2019).

Our small-RNA sequencing revealed fine regulation for both conserved and stress-responsive miRNAs across pollen developmental stages and related extracellular fractions (Fig. 2). The clustering and the overlap patterns across the samples supported the robustness of the datasets, while the strong induction of miRNAs by HS in hydrated pollen (HP) suggests that mature pollen is a major site of stress-responsive control. The identified miRNAs included elements present in conserved families involved in development and stress responses (e.g. miR156, miR160, miR167), as well as in growth and hormone signalling that might also participate in the defense against abiotic factors (e.g. miR158, miR162, miR168, miR399) (Xu *et al*., 2012; Ahmed *et al*., 2020). In addition, the presence of *Brassica*-specific miRNAs (those reported as Bra-miRN, Bna-miRN, Bju-miRN, Bol-miRN) further suggests lineage-specific regulatory mechanisms contributing to thermotolerance. Indeed, previous deep sequencing in *B. napus* has revealed 62 *Brassica*-specific miRNA candidates in addition to conserved ones (Xu *et al*., 2012).

Shared miRNAs between HP and germinated pollen (GP) indicated a preserved molecular core maintained across developmental stages and stress conditions (Dresselhaus and Franklin-Tong, 2013); in addition, the overlap between PS (e.g. Bju-miR6030a, Bju-miR160a, Bna-miR319a) and the extracellular vesicle-free (EVF) fraction (e.g. Bol-miRN302) profiles emphasized the functional continuity between the intracellular and secreted compartments. The stable expression of miR156/157 across all conditions highlights their role as a conserved regulatory axis essential for pollen physiology, orchestrating developmental and stress-responsive pathways including hormone signalling and the homeostasis of reactive oxygen species (Wang, 2014; Ahmed *et al*., 2019; Zuo *et al*., 2021).

The selection of the specific miRNAs for qPCR validation (Fig. 2D) was based on their established roles in plant developmental plasticity and stress adaptation. MiR159a and miR158a were selected because they are master regulators of gametophyte development: specifically, miR159 is known to control *GAMYB* transcription factors essential for male fertility (Millar and Gubler, 2005; Ma *et al*., 2017). Their modulation that we observed under HS, particularly the dramatic extracellular accumulation of miR159a, suggests a potential selective unloading mechanism. By exporting miR159a into the extracellular space (EVF and PS), the cell might effectively alleviate the repression of its *GAMYB* targets, thereby prioritizing the maintenance of reproductive development under thermal stress. Similarly, the involvement of miR161 and miR172 in phase transition and defense responses, often through the targeting of *AP2*-like genes (Aukerman and Sakai, 2003), suggests a selective re-routing of these signals during thermal stress. The significant reduction of miR172 in extracellular fractions under HS might reflect a strategic delay in developmental phase transitions, allowing the plant to redirect metabolic energy towards immediate heat-shock survival. Furthermore, the near-total disappearance of miR399 from the extracellular environment was particularly noteworthy. While it is a well-documented systemic signal for phosphate homeostasis (Chiou *et al*., 2006), its rapid depletion in our HS samples might indicate a broader role as a stress-responsive signal. Finally, the remarkable accumulation of miR289 across all fractions confirms its role as a robust marker for extracellular miRNA cargo in *Brassica* species, lending further weight to the reliability of our sequencing dataset and confirming the specificity of the heat-induced shifts observed for the other targets.

The selective enrichment of specific miRNAs in PS under HS suggests the existence of active sorting mechanisms rather than passive release. The stress-induced loading of miR160 and miR6030a into PS would support a model of heat-dependent remodelling of the vesicular cargo (Baldrich *et al*., 2019; He *et al*., 2021). The absence of certain miRNAs from PS-Ctrl but their accumulation under HS (Fig. 2C) reinforces the idea that the contribution of vesicular signalling varies depending on environmental pressures (Rutter and Innes, 2017; Makarova *et al*., 2021; Suanno *et al*., 2023). In addition, the presence of miR399 predominantly in the EVF fraction under control conditions could suggest preferential non-vesicular release in unstressed pollen (Bari *et al*., 2006; Rutter and Innes, 2017). Thus, the HS treatment appeared to alter EV biogenesis, lipid composition, and membrane dynamics, reshaping RNA-sorting mechanisms and favouring the encapsulation of specific miRNAs into vesicles (He *et al*., 2021).

The behaviour of Bju-miR399, a regulator of phosphate homeostasis (Bari *et al*., 2006), revealed a stress-dependent shift in its intracellular retention or extracellular release (Baldrich *et al*., 2019; He *et al*., 2021). Under control conditions, this miRNA was enriched in PS relative to the EVF fraction, indicating preferential vesicular accumulation. By contrast, when subjected to HS it was not selectively withheld within vesicles but instead secreted into the extracellular environment. Bju-miR399 was the only miRNA significantly down-regulated in PS under HS compared with control conditions, while concomitantly increasing in the EVF fraction, indicating that HS triggered its release from vesicles into the extracellular space and supporting a stress-dependent shift from vesicle-associated retention to non-vesicular secretion. Such a mechanism is in line with other models in which plants have been observed to redirect the activity of some miRNAs towards metabolic adaptation during alterations in nutrients or temperature (Bari *et al*., 2006; Baldrich *et al*., 2019; He *et al*., 2021). In parallel, HS promoted the preferential loading of other miRNAs, into PS, including Bju-miR160, Bju-miR6030a, and Bna-miR319a indicating selective changing of the vesicle cargo. Conversely, miRNAs that were abundant in HP under HS, such as Bju-miR9557 and Bol-miRN310b, appeared reduced in GP under HS, suggesting a reprogramming of regulatory priorities from developmental progression towards stress response (Ahmed *et al*., 2019; Keller *et al*., 2020; Zuo *et al*., 2021). This inversion of expression patterns reflects the activation of compensatory circuits aimed at maintaining reproductive function even under adverse conditions.

The shared presence of miRNAs such as Bol-miRN302, Bju-miR390a, and Bna-miRN297a across GP, PS, and EVF under both control and HS conditions indicated that germinating pollen continuously produced and exported a conserved set of miRNAs, potentially contributing to pollen–pistil or pollen–pollen communication. Taken together, these results support a model in which heat stress dynamically reshapes miRNA secretion pathways, altering the balance between vesicle-mediated and non-vesicular export in order to fine-tune adaptive responses during reproductive events. The enrichment of miR160 and miR319a in PS under HS suggests a selective release of regulatory signals. Indeed, both miRNAs, although originally linked to vegetative development, have been found to be abundant in pollen and are able to play key roles in regulating auxin responses and TEOSINTE BRANCHED 1/CYCLOIDEA/PROLIFERATING CELL FACTOR (TCP) transcription factors during gametophyte development (Chambers and Shuai, 2009; Grant-Downton *et al*., 2009).

For many miRNAs there is currently little or no functional information available, as they have only recently been predicted from genome analyses or are poorly characterized. Nonetheless, their occurrence across our HP, GP, EVF, and PS samples highlights previously unknown regulatory pathways in *B. napus*, opening new perspectives for future studies on their functional roles in pollen development and stress adaptation.

The prediction of developmental transcription factors (SPL, AP2, HD-ZIP, and MYB) as targets of the miRNAs that we identified (Table 1; Supplementary Dataset S2) is consistent with their established roles in reproductive development, where the miR156–miR172 module orchestrates phase transitions and floral-organ identity, and supports a specific function of these miRNAs in fine-tuning male gametophyte maturation (Jung *et al*., 2007; Wu *et al*., 2009).

The identification of hormone-related targets, particularly ARFs and isopentenyl transferases, demonstrates that auxin and cytokinin signalling, which are already associated with pollen differentiation and germination (Miyawaki *et al*., 2004; Grant-Downton *et al*., 2009; Borges *et al*., 2011), are tightly regulated at the post-transcriptional level by miRNAs.

The targeting of cytoskeletal regulators such as villins and myosins suggests that miRNAs might also modulate vesicle budding and pollen-tube elongation, processes that require changes in actin dynamics and rearrangements of microtubules (Cheung and Wu, 2008).

The predicted effects of pollen miRNAs on lipid-related transcripts, including lipases and acyltransferases that are typically involved in lipid metabolism and in the formation of protective pollen coat layers, were consistent with our lipidomics data. This supports the hypothesis of a coordinated regulation of membrane composition and vesicle stability (Upchurch, 2008; Narayanan *et al*., 2016). In addition, lipid remodelling enzymes and GPI-anchor proteins have been linked to EV membrane biogenesis, modulating membrane fluidity and microdomain organization during EV formation (Record *et al*., 2018). The identification of EV-related proteins among the miRNA predicted targets indicates that EV production is not a random phenomenon in plants, but a precise signalling system mediated by several components that include miRNAs. Membrane transporters, including ABC transporters and sucrose symporters, are frequently detected in plant EV proteomes and are thought to contribute both as cargo and as structural elements (Rutter and Innes, 2018; De Palma *et al*., 2020). Taken together, these observations suggest that miRNAs might indirectly shape the composition and influence the functionality of plant EVs by targeting proteins involved in membrane transport, cytoskeletal dynamics, and lipid metabolism.

The presence of stress-associated targets, including NLR immune receptors and ubiquitin ligases, implies that pollen miRNAs might coordinate plant immunity and reproductive development, especially during the phase of recognition between pollen and stigma. This aligns with evidence from Arabidopsis demonstrating that miRNAs and siRNAs post-transcriptionally silence NLR genes for homeostasis of immunity (Borrelli *et al*., 2018; López-Márquez *et al*., 2021) and that ubiquitin ligases regulate NLR receptor stability in plant innate immunity (Ortiz-Morea *et al*., 2022).

Our enrichment analysis revealed that miRNAs integrate developmental, structural, and metabolic programs in *B. napus* pollen (Fig. 3). Their strong representation in pollen confirms a fundamental role for them in male gametophyte differentiation and reproductive organ morphogenesis (Grant-Downton *et al*., 2009), while their association with elements from vesicle compartments (Golgi, endosome, vacuolar membrane) could provide corroboration that the transport of small RNAs in EVs plays a key function in pollen–stigma communication (Regente *et al*., 2017; Rutter and Innes, 2017). The identification of cytoskeletal- and microtubule motor-related categories further points to miRNA-mediated regulation of vesicle trafficking (van Niel *et al*., 2018; He *et al*., 2021). The enrichment in lipid transport terms together with the reduction in VLCFAs observed in our lipidomic analysis confirms a significant miRNA-dependent remodelling of membranes to sustain vesicle integrity under stress (Upchurch, 2008; Narayanan *et al*., 2016).

Collectively, these findings reinforce the role of miRNAs as central hubs in stress-responsive gene networks, extending beyond their established functions in developmental regulation and consistent with previous evidence from pollen studies in Arabidopsis (Grant-Downton *et al*., 2009; Wang, 2014; Zuo *et al*., 2021). It has been suggested that miRNA activity is dynamic in pollen development: first they regulate transcription factors, while in later stages they increasingly redirect to transposable elements, thereby coordinating developmental control and genome stability (Oliver *et al*., 2022). Our network analysis reinforces this view (Fig. 3C), revealing highly connected modules linking morphogenesis, hormone regulation, and reproductive development, while stress- and defense-related terms (although peripheral) appeared as complementary nodes.

Collectively, all these findings demonstrate evolutionary conserved functions for miRNAs during pollen germination, namely support for reproductive morphogenesis and remodelling in response to environmental stressors.

## Conclusion

Our findings demonstrate that elevated temperatures trigger a marked remodelling of the reproductive physiology of *B. napus*, affecting pollen productivity and germinability while reshaping its molecular secretome. Heat stress uncoupled pollen morphology and viability from reproductive output, markedly reducing pollen production while increasing pollensome nanoparticle abundance. These coordinated shifts indicate that thermal stress perturbs not only membrane dynamics and lipid-mediated signalling, but also the small-RNA regulatory circuits that are essential for pollen function. The identification of 61 differentially expressed miRNAs highlights a precise epigenetic strategy to manage the thermal challenge. By targeting pathways related to SPL transcription factors, auxin signalling, and cytoskeletal dynamics, these miRNA modules orchestrate the structural and hormonal shifts necessary for pollen-tube growth. The male gametophyte thus emerges as a particularly sensitive stage, underscoring a major vulnerability for crop productivity in a warming climate. By pinpointing stress-responsive lipids and miRNAs, this study provides molecular entry points and promising markers for screening natural variation and identifying genotypes with enhanced reproductive stability under heat stress. A natural progression of this work would involve the functional characterization of the identified components, with a specific focus on the experimental validation of the miRNA-target regulatory networks. Integrating such mechanistic insights into breeding frameworks will be essential to fully decipher the stress-response signals transported by extracellular fractions and should contribute to the development of *B. napus* varieties with improved tolerance to elevated temperatures.

## Supplementary Material

erag303_Supplementary_Data

## Data Availability

All data supporting the findings of this study are available within the paper and within its supplementary materials published online. Additional information and data are available from the corresponding author, Angelo Gismondi, upon request.

## Supplementary data

The following supplementary data are available at *JXB* online.

Dataset S1. Raw data from the GC-MS analysis.

Dataset S2. Output of the raw data from the bioinformatic prediction by psRNATarget.

Table S1. List of the expressed miRNAs identified by high-throughput sequencing.

Table S2. Percentage variation of miRNA abundance across the different pollen fractions under heat stress.
