# Supplementary material for: Heat stress-induced remodelling of lipid and microRNA networks in Brassica napus germinated pollen and pollen-derived small extracellular vesicles
Source: J Exp Bot. 2026 Jun 23;77(17):5936–55. doi: 10.1093/jxb/erag303 (PMC13577775; doi:10.1093/jxb/erag303)
Supplement: Supplementary file 1 [file erag303_supplementary_data.zip › JEXBOT317922-file001.pdf]

Supplementary Table S1. List of the expressed miRNAs. Nucleotide sequence and number of raw sequencing reads counted for each miRNA identified by High Throughput Sequencing analysis in the various samples are reported. Abbreviations: Ctrl, control plants; HS, heat stress; HP, hydrated pollen; GP, germinated pollen; PS, pollensomes; EVF, vesicle-free medium; Bju, *Brassica juncea*; Bna, *Brassica napus*; Bni, *Brassica nigra*; Bol, *Brassica oleracea*; Bra, *Brassica rapa*.

miRNA	Sequence (5'-3')	HP-Ctrl	GP-Ctrl	EVF-Ctrl	PS-Ctrl	HP-HS	GP-HS	EVF-HS	PS-HS
**Bra-miRN357a**	TGCGGAAGGATCATTGTCGTA	488028	606204	719940	577778	317144	513944	804842	726703
**Bju-miR158a**	TTTCCAAATGTAGACAAAGCA	157846	74388	2679	0	224970	82112	7581	29570
**Bju-miR400a**	TATGAGAGTATTATAAGTCAC	104218	44314	0	0	86644	67121	1223	0
**Bra-miRN328**	TTACTTATTGTCGGTTGACTTT	67077	176011	2381	0	151548	228712	6603	3584
**Bju-miR156b**	CTGACAGAAGAGAGTGAGCAC	53910	9410	63393	172840	50283	10399	56248	63620
**Bju-miR827a**	TTAGATGACCATCAACAAATA	33876	9162	0	0	40048	17152	0	0
**Bju-miR399**	TGCCAAAGGAGAGTTGCCCTG	12099	4581	893	76543	11347	6212	0	0
**Bju-miR397a**	TCATTGAGTGCAGCGTTGATGT	9932	2141	2976	0	6484	1688	2201	3584
**Bna-miRN289**	GGCGGGCTGCTTGAGCTGCA	9369	50139	115476	165432	23616	44703	60651	138889
**Bra-miRN351**	GCTTGTGGTTCGGACACTCT	8328	2490	2381	0	25650	3511	1956	6272
**Bol-miR168b**	TCGCTTGGTGCAGGTCGGGACT	7625	2241	2083	0	11442	1418	5136	5376
**Bra-miRN323**	TTGAAACTTTGATCTAGATC	7512	3734	0	0	9821	6280	0	0
**Bna-miRN298c**	TCGGGGATAGTTGGGAATGTGG	4643	1743	53869	0	3464	743	24700	1792
**Bra-miR9563**	AGCGGAATATAAGAACCCGTCTCT	4220	1494	0	0	4164	1080	0	0
**Bju-miR845**	CGGCTCTGATACCAGTTGATG	3208	1344	0	0	4577	1351	0	0
**Bra-miRN321**	ATAGAGGAAAAAATAGAGTTC	2589	846	0	0	2002	810	0	0
**Bol-miRN309a**	TCGAGAGTGGTTGGAAAGATT	2026	249	0	0	1303	743	0	0
**Bju-miR403a**	TTAGATTCACGCACAAACTCG	1885	697	0	0	2733	1013	0	0
**Bju-miR6030a**	TCCACCCATACCATACAGACCC	1857	597	0	0	1875	1283	0	2688
**Bju-miR5654a**	ATAAATCCCAAGCATCATCCA	1519	498	0	0	1589	1013	3913	0
**Bju-miRN271**	TTGGTAAGATTATGAGAGCATG	1407	349	0	0	1208	68	734	0
**Bju-miR160a**	TGTCTGGCTCCCTGTATGCCA	1125	448	0	0	2034	203	0	2688
**Bju-miR162a**	TCGATAAACCTCTGCATCCAG	1097	597	0	0	2002	1080	0	0
**Bna-miRN280a**	TTGAAAGTCGTCCATGATAAG	1041	349	0	0	731	405	0	0
**Bol-miR157**	TTGACAGAAGATAGAGAGCACT	1041	349	3274	7407	1017	405	2446	2688
**Bra-miRN354**	AATGGAGTGGGAAATGGAGTA	929	647	298	0	922	270	0	0
**Bna-miRN287a**	TCGACGAGAGGAACACGGTGG	900	349	0	0	699	68	2201	0
**Bju-miR172a**	GGAATCTTGATGATGCTGCAT	816	548	1190	0	540	743	0	0
**Bol-miRN312**	ACAGGTAGAATTGTCGGCTG	732	249	298	0	795	135	0	0
**Bol-miRN304b**	AGGGGATGATTGGTTGTGGCTG	675	149	8929	0	254	68	2446	0
**Bol-miR167c**	TGAAGCTGCCAGCATGATCTAT	619	149	0	0	731	270	0	0
**Bra-miRN367**	ATCTTGTCGGAGTTTATGATC	563	548	0	0	636	675	734	0
**Bju-miR165i**	TCGGACCAGGCTTCATTCCCCTC	506	249	5952	0	699	203	734	7168
**Bol-miRN313**	ATAAGAGCATGATTATTGGGGG	422	100	1786	0	191	135	0	0
**Bju-miR170a**	TGATTGAGCCGCGTCAATATC	394	100	0	0	159	675	489	0
**Bra-miRN377a**	TGGATATGATGAAATGGCATA	394	0	0	0	95	0	0	0
**Bna-miRN298a**	GGGATAGTTGAGAATGTGGC	338	50	1488	0	350	135	0	0
**Bju-miR171e**	TTGAGCCGTGCCAATATCACG	310	149	0	0	64	68	0	0
**Bju-miRN272**	GTTATTCTATTCCACCTCTT	310	349	0	0	286	338	0	0

**Bna-miR399c**	TGCCAAAGGAGATTTGTCCGG	310	100	0	0	445	68	0	0
**Bna-miRN298b**	GGGGGATAGTTGGGACTGTGGC	310	50	893	0	95	0	1223	0
**Bna-miR1885**	CATCAATGAAAGGTATGATTCC	253	0	0	0	32	135	0	0
**Bni-miRN300**	TGTGTTGTGATGATAATCTGA	253	0	0	0	318	68	0	0
**Bra-miR9552b**	CGGTGATGAAGAAGCTAGCGC	253	0	0	0	159	68	0	0
**Bra-miRN316**	TGAATGTCTTTCTCTTCATC	253	50	0	0	159	135	0	0
**Bna-miRN277a**	TCCATCGATCGAGTGACGGCTT	225	0	0	0	127	68	0	0
**Bna-miRN283**	ATACGTGTTGAGTCTTGAGTGG	197	0	0	0	191	0	1223	0
**Bol-miR9410**	ACTTAATTATAAGTCGTCTGG	197	50	0	0	32	0	0	0
**Bol-miR9411**	TACTGGACGACTTACACGGAAG	197	0	0	0	95	68	0	0
**Bol-miRN308**	AGTGGTAAAGAGGTTGCGGCTG	197	0	0	0	159	0	978	0
**Bra-miR5714**	TTTCTTGAGGTTGTAGAGTCT	197	0	0	0	413	135	0	0
**Bju-miR159a**	TTTGGATTGAAGGGAGCTCTA	169	448	595	0	64	203	5625	1792
**Bju-miR6028**	TGGAGAGTAAAGACATTCAGAT	169	50	0	0	64	68	0	0
**Bna-miR319a**	TTGGACTGAAGGGAGCTCCCTT	169	199	0	0	413	203	0	896
**Bra-miRN329**	TTGTTCTTCAATATGTACTTG	169	149	0	0	350	68	0	0
**Bju-miRN265**	AACGAACGAGGAAGAAGAGCT	141	0	0	0	64	0	0	0
**Bra-miRN317**	TTTTTCCTCGGAATTTCCTCG	141	100	0	0	381	68	0	0
**Bra-miRN334**	TACAAATCCTCCAACTTTTCTT	141	100	0	0	0	0	0	0
**Bju-miR6029**	TGGGGTTGTGATTTCAGGCTT	113	50	0	0	318	203	0	0
**Bna-miRN294**	TTGTGATCCGAGTGGAGACAA	113	299	298	0	350	473	0	0
**Bra-miRN339**	CCAGTAGTTCGTTAGGTTTGAC	113	50	0	0	445	135	0	0
**Bju-miR161**	TCAATGCACTGAAAGTGACTA	84	50	0	0	191	473	0	0
**Bra-miRN332**	TATTGCGTGCTGTTTCATGGCT	84	0	0	0	159	68	0	0
**Bju-miR9557**	TTTTGCGTTTCAACTCGGTCC	28	50	0	0	159	0	0	0
**Bna-miRN286**	TTTGTTGGACTTGCTATGGC	28	0	0	0	191	0	0	0
**Bna-miRN297a**	TAGAGAGAGAGAGTCGGCGG	28	50	3274	0	0	0	2446	0
**Bol-miRN302**	GGAGGAGAGAGAGTCGGCGG	28	0	3274	0	95	0	2201	0
**Bol-miRN310b**	GAATGAGTCTTCCATGTGGC	28	50	0	0	191	0	0	0
**Bra-miRN337**	ACCAAATGATGGTCATTATTTA	28	100	0	0	159	68	0	0
**Bju-miR390a**	AAGCTCAGGAGGGATAGCGCC	0	0	2381	0	64	0	1467	2688

Supplementary Table S2. Percentage variation of miRNA abundance under heat stress. This table reports the percentage change in raw sequencing reads for each identified miRNA across different pollen fractions (HP, GP, EVF, and PS) exposed to heat stress (HS) compared to the respective control (Ctrl) equivalents. Color-coding has been applied to facilitate data interpretation: ochre indicates an increase in abundance (positive percentage, higher values in HS), while blue represents a decrease (negative percentage, lower values in HS). A dash was reported when miRNAs were not detected in the Ctrl or HS samples. Abbreviations: HP, hydrated pollen; GP, germinated pollen; PS, pollensomes; EVF, vesicle-free medium; Bju, *Brassica juncea*; Bna, *Brassica napus*; Bni, *Brassica nigra*; Bol, *Brassica oleracea*; Bra, *Brassica rapa*.

microRNA	HP-HS	GP-HS	EVF-HS	PS-HS
**Bra-miRN357a**	-35	-15	12	26
**Bju-miR158a**	43	10	183	-
**Bju-miR400a**	-17	51	-	-
**Bra-miRN328**	126	30	177	-
**Bju-miR156b**	-7	11	-11	-63
**Bju-miR827a**	18	87	-	-
**Bju-miR399**	-6	36	-100	-100
**Bju-miR397a**	-35	-21	-26	-
**Bna-miRN289**	152	-11	-47	-16
**Bra-miRN351**	208	41	-18	-
**Bol-miR168b**	50	-37	147	-
**Bra-miRN323**	31	68	-	-
**Bna-miRN298c**	-25	-57	-54	-
**Bra-miR9563**	-1	-28	-	-
**Bju-miR845**	43	1	-	-
**Bra-miRN321**	-23	-4	-	-
**Bol-miRN309a**	-36	198	-	-
**Bju-miR403a**	45	45	-	-
**Bju-miR6030a**	1	115	-	-
**Bju-miR5654a**	5	103	-	-
**Bju-miRN271**	-14	-81	-	-
**Bju-miR160a**	81	-55	-	-
**Bju-miR162a**	82	81	-	-
**Bna-miRN280a**	-30	16	-	-
**Bol-miR157**	-2	16	-25	-64
**Bra-miRN354**	-1	-58	-100	-
**Bna-miRN287a**	-22	-81	-	-
**Bju-miR172a**	-34	36	-100	-
**Bol-miRN312**	9	-46	-100	-
**Bol-miRN304b**	-62	-54	-73	-

**Bol-miR167c**	18	81	-	-
**Bra-miRN367**	13	23	-	-
**Bju-miR165i**	38	-18	-88	-
**Bol-miRN313**	-55	35	-100	-
**Bju-miR170a**	-60	575	-	-
**Bra-miRN377a**	-76	-	-	-
**Bna-miRN298a**	4	170	-100	-
**Bju-miR171e**	-79	-54	-	-
**Bju-miRN272**	-8	-3	-	-
**Bna-miR399c**	44	-32	-	-
**Bna-miRN298b**	-69	-100	37	-
**Bna-miR1885**	-87	-	-	-
**Bni-miRN300**	26	-	-	-
**Bra-miR9552b**	-37	-	-	-
**Bra-miRN316**	-37	170	-	-
**Bna-miRN277a**	-44	-	-	-
**Bna-miRN283**	-3	-	-	-
**Bol-miR9410**	-84	-100	-	-
**Bol-miR9411**	-52	-	-	-
**Bol-miRN308**	-19	-	-	-
**Bra-miR5714**	110	-	-	-
**Bju-miR159a**	-62	-55	845	-
**Bju-miR6028**	-62	36	-	-
**Bna-miR319a**	144	2	-	-
**Bra-miRN329**	107	-54	-	-
**Bju-miRN265**	-55	-	-	-
**Bra-miRN317**	170	-32	-	-
**Bra-miRN334**	-100	-100	-	-
**Bju-miR6029**	181	306	-	-
**Bna-miRN294**	210	58	-100	-
**Bra-miRN339**	294	170	-	-
**Bju-miR161**	127	846	-	-
**Bra-miRN332**	89	-	-	-
**Bju-miR9557**	468	-100	-	-
**Bna-miRN286**	582	-	-	-
**Bna-miRN297a**	-100	-100	-25	-
**Bol-miRN302**	239	-	-33	-
**Bol-miRN310b**	582	-100	-	-
**Bra-miRN337**	468	-32	-	-
**Bju-miR390a**	-	-	-38	-
